# Profiling of miRNAs and target genes related to cystogenesis in ADPKD mouse models

**DOI:** 10.1038/s41598-017-14083-8

**Published:** 2017-10-26

**Authors:** Yu Mi Woo, Do Yeon Kim, Nam Jin Koo, Yong-Min Kim, Sunyoung Lee, Je Yeong Ko, Yubin Shin, Bo Hye Kim, Hyowon Mun, Seonju Choi, Eun Ji Lee, Jeong-Oh Shin, Eun Young Park, Jinwoong Bok, Jong Hoon Park

**Affiliations:** 1 Department of Biological Science, Sookmyung Women’s University, Seoul, 04310 Republic of Korea; 2 Korean Bioinformation Center, Korea Institute of Bioscience and Biotechnology, Daejeon, 34141 Republic of Korea; 3 Department of Anatomy, Yonsei University College of Medicine, Seoul, 03722 Republic of Korea; 4 Departments of Anatomy and Otorhinolaryngology, and BK21 PLUS Project for Medical Science, Yonsei University College of Medicine, Seoul, 03722 Republic of Korea

## Abstract

Autosomal polycystic kidney disease (ADPKD) is a common inherited renal disease characterized by the development of numerous fluid-filled cysts in both kidneys. We investigated miRNA-mediated regulatory systems and networks that play an important role during cystogenesis through integrative analysis of miRNA- and RNA-seq using two ADPKD mouse models (conditional *Pkd1-* or *Pkd2*-deficient mice), at three different time points (P1, P3, and P7). At each time point, we identified 13 differentially expressed miRNAs (DEmiRs) and their potential targets in agreement with cyst progression in both mouse models. These targets were involved in well-known signaling pathways linked to cystogenesis. More specifically, we found that the actin cytoskeleton pathway was highly enriched and connected with other well-known pathways of ADPKD. We verified that miR-182-5p regulates actin cytoskeleton rearrangement and promotes ADPKD cystogenesis by repressing its target genes—*Wasf2*, *Dock1*, and *Itga4*—*in vitro* and *in vivo*. Our data suggest that actin cytoskeleton may play an important role in renal cystogenesis, and miR-182-5p is a novel regulator of actin cytoskeleton and cyst progression. Furthermore, this study provides a systemic network of both key miRNAs and their targets associated with cyst growth in ADPKD.

## Introduction

Autosomal dominant polycystic kidney disease (ADPKD) is the most common inherited renal disorder, characterized by the development of numerous fluid-filled cysts. It is caused by mutations in either *PKD1* (approximately 85% of cases) or *PKD2* (approximately 15% of cases)^1^. Reduced dosage of Polycystin (PC) 1, the protein encoded by the *PKD1* gene, can lead to the clinical features of ADPKD^2^. Recently, whole-exome sequencing identified mutation in GANAB, encoding glucosidase II subunit α (GIIα), cause ADPKD and liver disease^3^. Cyst initiation and enlargement arise from disturbances in the balance between tubular cell proliferation and apoptosis, alterations in the cell polarity of membrane proteins, extracellular matrix defects, abnormal fluid secretion, and abnormal ciliary function, which induces kidney enlargement and interstitial fibrosis^4,5^. Although many pathways are involved in ADPKD, including Ca^2+^, cAMP, Wnt, JAK2/STAT1/p21, ERK, and mTOR^6–10^, an assumption questioned by several recent studies suggests an unrevealed signaling pathway to be the fatal cause^11–15^. Indeed, the severity of renal disease is highly variable even within the same family and among patients with ADPKD. Therefore, increasing evidence suggests a critical role for epigenetic modifications as another risk factor in cystogenesis.

Cell migration plays a key role in many physiological and pathological processes. Migratory stimuli induce a multistep cell migration process, which first involves the acquisition of a characteristic polarized morphology toward the stimulus, called the leading edge. At the leading edge, flat membrane protrusions, lamellipodia, and finger-like protrusions, filopodia, are formed by actin polymerization^16^. Activated N-WASP/WAVE and Arp2/3 proteins induce nucleation of a branched actin network at the lamellipodium^17^. In particular, both PC1 and Pacsin2 co-localize on the lamellipodia of migrating kidney epithelial cells, and are required for N-Wasp/Arp2/3-dependent actin remodeling as members of the same protein complex^18^. However, direct mechanisms that cause defective actin cytoskeleton function during cyst formation and enlargement in PKD disease models have not been elucidated.

MicroRNAs (miRNAs) are small, regulatory, non-coding RNAs that negatively regulate multiple protein-coding genes by binding seed sequences in their 3′UTRs^19,20^. Recent studies have revealed that miRNAs are closely associated with cystogenesis in ADPKD^21^. Mice lacking Dicer, a key enzyme in miRNA biogenesis, in renal tubules and collecting ducts showed defects in kidney tubule maturation such as hydronephrosis, hydroureter, and cyst formation^22^. Additionally, the upregulation of several miRNAs, including the miR-17/92 miRNA cluster and miR-21, accelerates renal cyst development in ADPKD mouse models^23,24^.

Constitutive *Pkd1*- or *Pkd2*-targeted knockout mouse models show embryonic lethality with renal cysts, liver cysts, cardiovascular defects, and dysregulated skeletal development^25^. However, *Pkd1* or *Pkd2* conditional knockout mice, which are kidney-specifically deficient, are born normally and show rapid cyst formation from postnatal day 1 (P1), accompanied by increased cell proliferation by abnormal activation of the MAPK/ERK pathway^26^. Herein, we used kidney collecting-duct-specific Cre-expressing mice for *Pkd1* or *Pkd2* conditional inactivation.

In this study, we investigated miRNA profiles associated with severity of cyst development in the ADPKD models of *Pkd1* or *Pkd2* conditional knockout mice. From the parallel analysis of miRNA-sequencing and RNA-sequencing, we identified key miRNAs and their targets that are involved in the actin cytoskeleton pathway, a novel cystogenesis-related signaling mechanism.

## Results

### Cyst formation derived from HoxB7-cre-mediated inactivation of the *Pkd1*^flox^ allele

**Figure 1:**
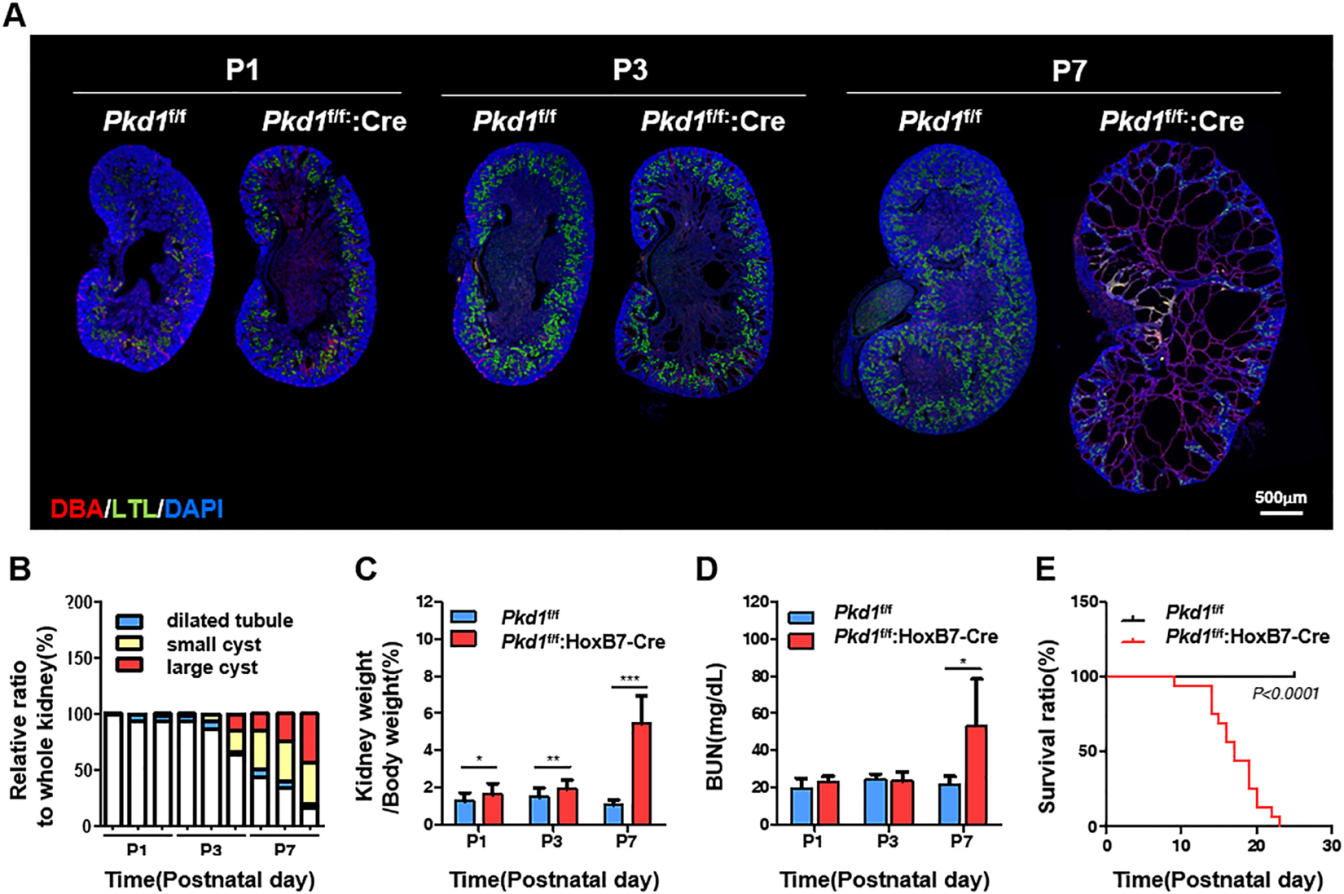
Characterization of mice with kidney collecting duct-specific *Pkd1* knockout. (**A**) The whole kidney sections from mice at postnatal day 1 (P1), P3, and P7 were stained with the collecting duct-specific marker *Dolichos biflorus agglutinin* (DBA, red) and the proximal tubule marker *Lotus tetragonolobus* lectin (LTL, green). Nuclei were counterstained with DAPI (blue). (**B**) The areas of cysts derived from collecting ducts were measured and classified by cyst sizes: non-cystic area, dilated tubules (<1 × 10^5^ μm^2^), small cysts (1 × 10^5^–10^6^ μm^2^), and large cysts (>1 × 10^6^ μm^2^). (**C**) Kidney/body weight ratio and (**D**) serum urea nitrogen (BUN) concentration in *Pkd1* ^f/f^ versus *Pkd1* ^f/f^:HoxB7-Cre mice. Data are shown as mean ± SD; n ≥ 10 for each time point; *P < 0.05; **P < 0.01; ***P < 0.001. (**E**) Survival rate curves of *Pkd1* ^f/f^ versus *Pkd1* ^f/f^:HoxB7-Cre mice. n ≥ 10.

To investigate detailed phenotypic changes resulting from the collecting-duct-specific inactivation of either *Pkd1* or *Pkd2*, kidney samples were collected and cyst formation and renal function were investigated at postnatal day 1 (P1), P3, and P7 in each rodent model. Immunofluorescence showed that most cysts were derived from the collecting duct. To identify the origin of renal tubular cysts, nephron segment-specific markers were used. In *Pkd1*- or *Pkd2*-deficient mice, all dilated tubules and cysts lining epithelial cells stained positively for *Dolichos biflorus* agglutinin (DBA), which is specifically expressed in the collecting duct. In contrast, none of the cysts stained positively for *Lotus tetragonolobus* lectin (LTL), a proximal tubule marker (Supplementary Fig. S1). Several dilated tubules were observed in both kidneys from *Pkd1* ^f/f^:HoxB7-cre mice at P1 and occasional cyst formation was observed starting at P3 in these mice. After P3, cyst size and kidney volume were rapidly increased. In particular, loss of renal function was observed in P7 *Pkd1* ^f/f^:HoxB7-cre mice (Fig. 1A–D). *Pkd2* ^f/f^:HoxB7-cre mice also showed phenotypic changes similar to those in *Pkd1* ^f/f^:HoxB7-cre mice (Supplementary Fig. S2), except for survival rate, which indicated longer survival in *Pkd2* ^f/f^:HoxB7-cre mice than in *Pkd1* ^f/f^:HoxB7-cre mice (Fig. 1E). These data suggested that conditional inactivation of *Pkd1*- or *Pkd2* may share common cyst formation processes in early stages, but different cyst expansion processes in later stages.

### Integrated analysis of miRNA-seq and mRNA-seq

**Figure 2:**
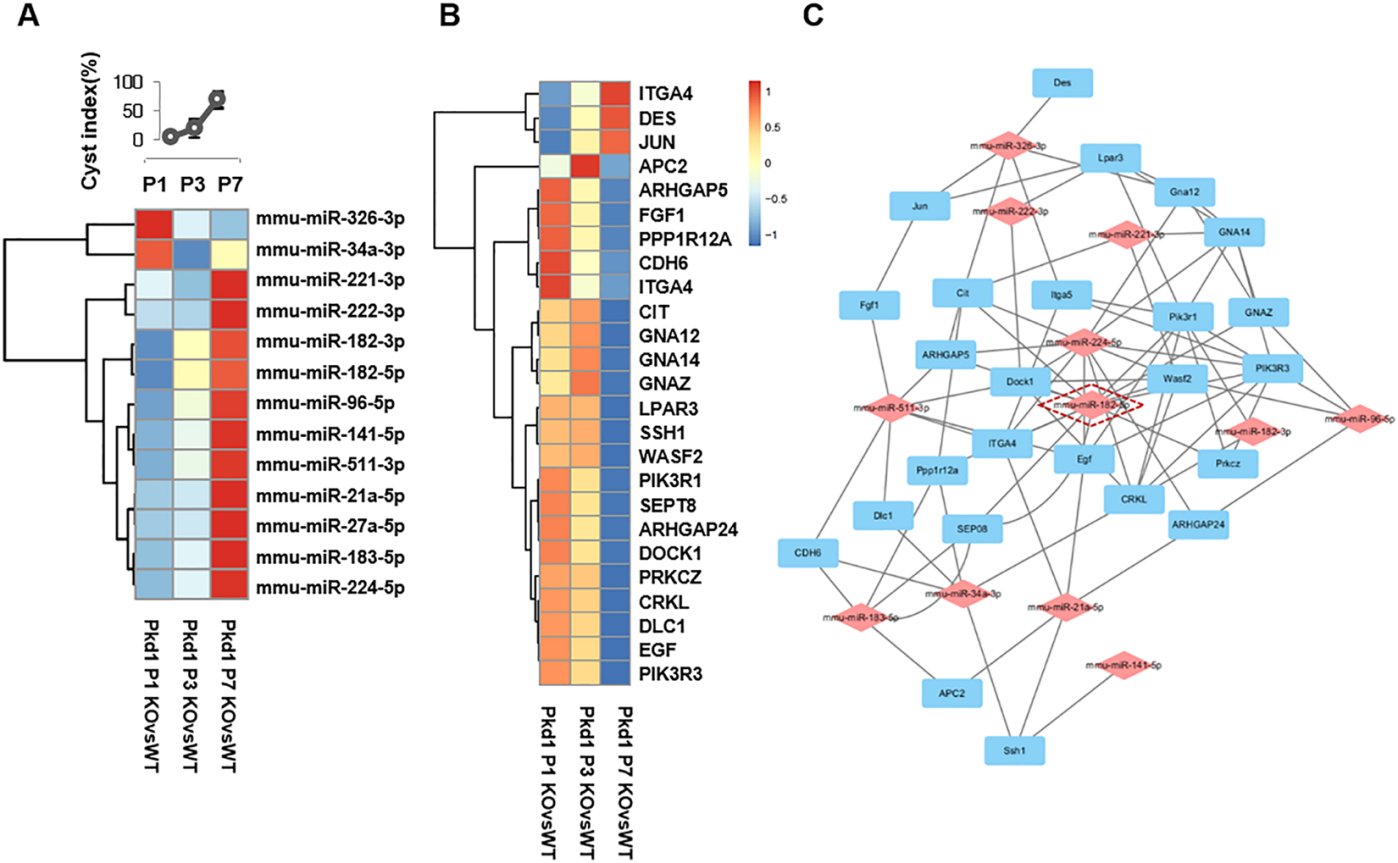
Correlation between cystogenesis-regulated miRNAs and candidate target mRNAs to the actin cytoskeleton. (**A**) Heat map of 13 differentially expressed miRNAs (DEmiRs) which were commonly changed in kidneys of both *Pkd1* ^f/f^:HoxB7-Cre and *Pkd2* ^f/f^:HoxB7-Cre mice for each time point. (**B**) Heat map displaying the differentially expressed mRNAs which are candidate targets of the 13 common DEmiRs from both mouse models and related with actin cytoskeleton signaling in *Pkd1* ^f/f^:HoxB7-Cre mice. (**C**) Interactive network between 13 DEmiRs and differentially expressed mRNAs associated with the actin cytoskeleton signaling pathway.

Given the phenotypic changes, both miRNA-seq and RNA-seq analyses were carried out using kidney tissues from the two mouse models at the indicated time points (P1, P3, and P7, n = 3). An integrative analysis of both types of NGS data was performed as shown in Supplementary Fig. S3. To profile transcripts and their expressions, we measured read counts instead of RPKM^27^ or FPKM^28^ values, because this was a more accurate measure for comparing differences between the mouse models at indicated time points^29^. miRNA expression was also measured based on known sequences. The read count values for each mouse model were normalized and used for further analysis. To enhance the candidate pools of mRNAs and miRNAs, mRNAs and miRNAs showing negative binomial distributions were identified independently and correlation analysis between mRNAs and miRNAs were performed. First, the most popular two methods (edgeR^30^ and Deseq. 2^31^) were performed in both wild-type (WT; *Pkd1* ^f/f^ and *Pkd2* ^f/f^) and knockout (KO; *Pkd1* ^f/f^:HoxB7-cre and *Pkd2* ^f/f^:HoxB7-cre) models to identify significantly differentially expressed mRNAs and miRNAs. For RNA-seq analysis, 3,040 transcripts in *Pkd1* ^f/f^:HoxB7-cre mice and 2,470 transcripts in *Pkd2* ^f/f^:HoxB7-cre mice were differentially expressed at the indicated time points (FDR < 0.05). In addition, we also identified 1,297 commonly regulated transcripts in both mouse models. For miRNA analysis, 243 miRNAs were differentially expressed, while 130 miRNAs were common in both mouse models (Supplementary Tables S1, 2, 3 and 4). Among the common miRNAs, 13 miRNAs, including 11 upregulated and two downregulated miRNAs (Fig. 2A), were selected based on expression pattern comparison at each time point. Second, correlation analysis was carried out using 13 DEmiRs and differentially expressed genes (DEGs) in both mouse models. Then, pathway analysis was performed and well-known pathways involved in cystogenesis including ERK/MAPK, AKT/mTOR, JAK/STAT, Wnt/β-catenin, PCP pathway, cell cycle, and TGF-β signaling pathways were identified along with novel pathways, such as the actin cytoskeleton pathway. miRNAs in the actin cytoskeleton pathway were highly enriched and delicately connected with other well-known signaling pathways (Supplementary Tables S5 and S6). Expression of candidate DEmiR targets associated with the actin cytoskeleton were gradually changed along with cyst progression in *Pkd1* ^f/f^:HoxB7-cre mice (Fig. 2B). Remarkably, miR-182-5p had multiple candidate targets involved in actin cytoskeleton signaling (Fig. 2C). The data indicated that 13 miRNAs were commonly associated with cystogenesis-related pathways, although they may target different mRNAs in two mouse models of ADPKD.

### Validation of key miRNAs and candidate target genes

**Figure 3:**
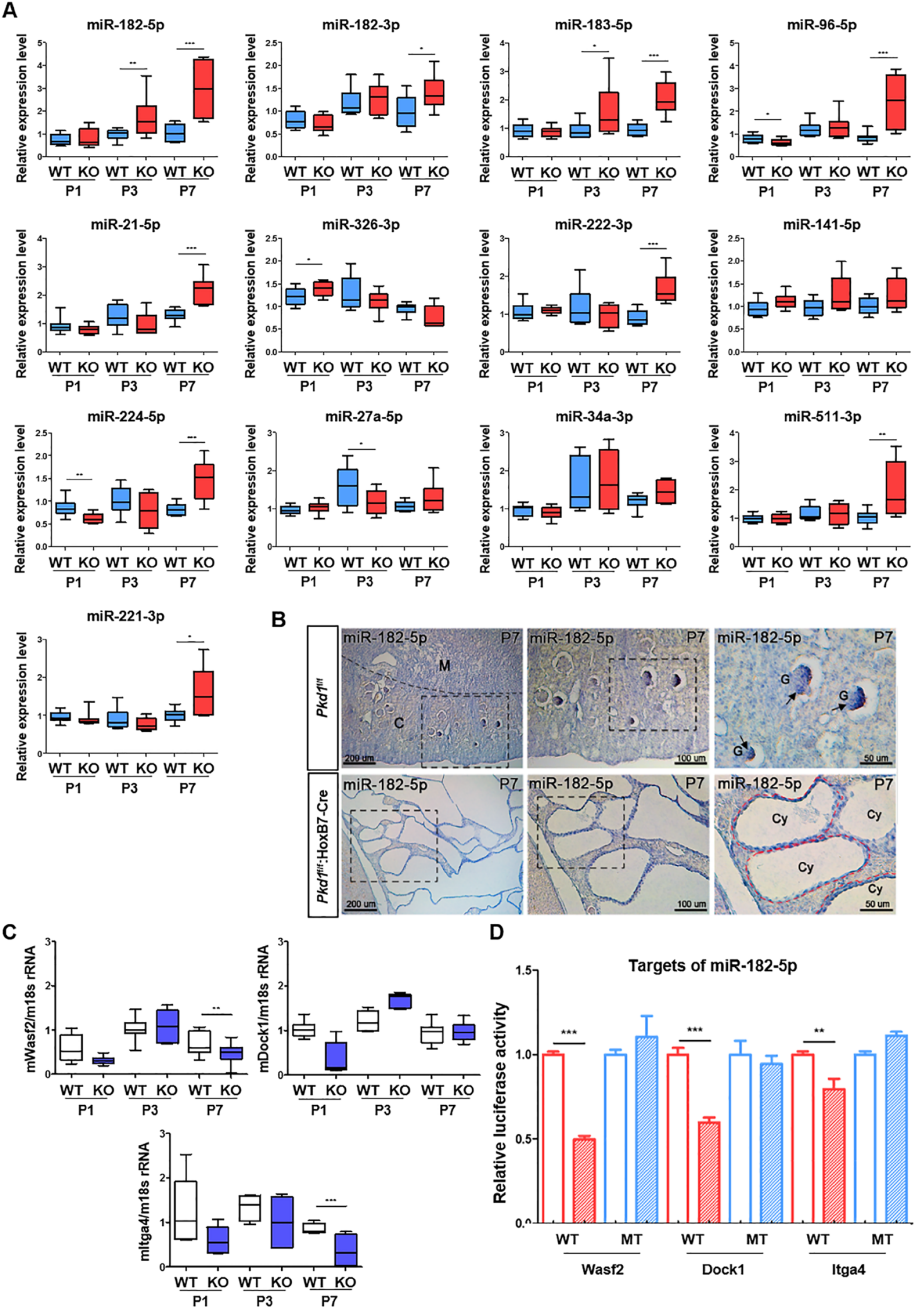
Expression validation of key miRNAs and target mRNAs in kidney tissues of *Pkd1* conditional knockout mice. (**A**) The expression level of 13 key miRNAs and (**C**) candidate target mRNAs of miR-182-5p were confirmed in kidneys of *Pkd1* ^f/f^:HoxB7-Cre at P1, P3, and P7 using quantitative real-time RT-PCR. Target mRNAs included genes related with cystogenesis and the actin cytoskeleton (*Wasf2*, *Dock1*, *Itga4*). *RNU6* and 18*s rRNA* were used as internal controls for miRNA and mRNA, respectively. The experiment was performed in triplicate. n ≥ 3 for each time point. (**B**) Alteration of miR-182-5p expression in *Pkd1* ^f/f^:HoxB7-Cre mouse kidney at P7. At P7, the expression pattern of miR-182-5p is detected by section *in situ* hybridization (ISH) in *Pkd1* ^f/f^ and *Pkd1* ^f/f^:HoxB7-Cre mice. M, medulla; C, cortex; G, glomerulus; Cy, cyst; black arrow, miR-182-expressing glomerulus; black dotted box, high magnification region; red dotted line, cyst lining epithelial cells. scale bars indicate left 200 µm; middle 100 µm; right 50 µm. (**D**) Relative 3′UTR luciferase activity of *Wasf2*, *Dock1*, *and Itga4* genes upon transfecting mIMCD cells with Negative Control mimic (NC mimic) or miR-182-5p mimic. Mutating the seed sequence of miR-182-5p induced rescued luciferase activity of the psiCHECK-2 vector. WT (wild type), MT (mutant type). Data are presented as mean ± SD of three independent experiment in triplicate. ***P < 0.001; **P < 0.01.

To validate the correlation analysis, expression levels of 13 key miRNAs were confirmed in the kidney tissues of *Pkd1* ^f/f^:HoxB7-cre mice using qRT-PCR. Most miRNAs, including miR-182-5p, showed a similar expression pattern between qRT-PCR and sequencing results (Fig. 3A). Interestingly, we observed that all miR-182 family members, miR-182-3p, miR-182-5p, miR-183-5p, and miR-96-5p, were significantly upregulated in P7 *Pkd1* ^f/f^:HoxB7-cre mice kidney tissues. Upon considering both small RNA-seq raw data and qRT-PCR, we concluded that miR-182-5p was the most suitable subject for future research. To identify alterations of spatiotemporal miR-182-5p expression in the mouse postnatal kidney, section miRNA *in situ* hybridization (ISH) was performed. Expression of miR-182-5p was detected in the *Pkd1* ^f/f^ medulla region and glomerulus. Interestingly, miR-182-5p-expressing cells were observed in the cuboidal epithelial lining cells of the cyst in *Pkd1* ^f/f^:HoxB7-Cre mice at P7 (Fig. 3B). miR-182-5p was localized in the overall kidney region at P1 and P3, including the medulla and cortex regions (Supplementary Fig. S4). The glomerulus showed especially strong miR-182-5p expression in P1 *Pkd1* ^f/f^ mice (Supplementary Fig. S4A–C). In the *Pkd1* ^f/f^:HoxB7-Cre mice, as expected, miR-182-5p was strongly expressed in the cyst-lining epithelial cells (Supplementary Fig. S4D–F and J–L). In *Pkd1* ^f/f^ at P3, interestingly, miR-182-5p expression was observed in the renal tubule domain, but the glomerulus and Bowman’s capsule cells did not exhibit miR-182-5p-expressing cells (Supplementary Fig. S4G–I). Candidate target mRNAs of miR-182-5p were downregulated in the kidney tissues of *Pkd1* ^f/f^:HoxB7-cre mice over time (Fig. 3C and Supplementary Fig. S5A). Furthermore, the expression of genes involved in ERK/MAPK and AKT/mTOR signaling pathways was increased; these pathways regulate cell proliferation in ADPKD (Supplementary Fig. S6A and C). Indeed, when we examined cell proliferation at the three time-points by ki67 staining, proliferating cells were markedly observed in cyst-lining cells of both KO mouse models, but not in WT kidney tissues (Supplementary Fig. S6B,D and E). In particular, in order to investigate the link between miR-182-5p and targets involved in actin cytoskeleton signaling as a novel cystogenesis pathway, a 3′UTR luciferase assay for miR-182-5p was performed. We found that miR-182-5p directly bound to the 3′UTR of these candidate target mRNAs related to actin cytoskeleton, negatively regulating their translation (Fig. 3D and Supplementary Fig. S5B). Therefore, our results were consistent with those of previous studies and helped us identify miRNAs that bridge ADPKD actin cytoskeleton defects and cystogenesis.

### Cyst formation upon actin cytoskeleton defects

**Figure 4:**
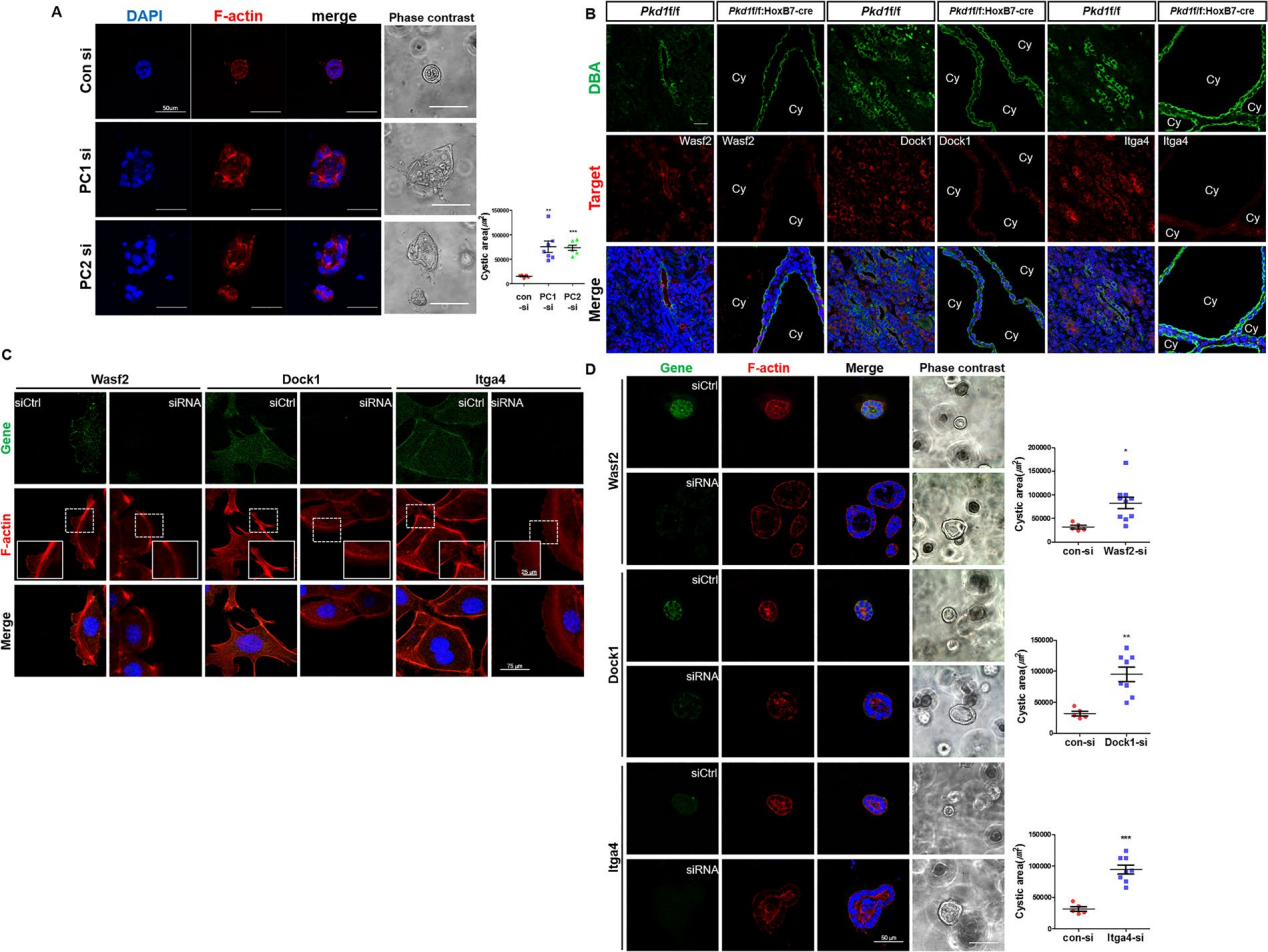
Defects in the actin cytoskeleton promote cyst development and growth. (**A**,**D**) *Pkd1, Pkd2, Wasf2*, *Dock1*, and *Itga4* siRNA-transfected mIMCD cells showed formed larger cysts than control siRNA-transfected mIMCD cells did. After 6 days of mIMCD culture in matrigel, representative images were obtained and cyst lumen sizes were randomly measured in mIMCD spheroids. Each spheroid was stained with rhodamine-labeled phalloidin (red) for F-actin. Nuclei were counterstained with DAPI (blue). (**B**) Expression of Wasf2, Dock1, and Itga4 protein (red) was analyzed by immunofluorescence in kidneys of *Pkd1* ^f/f^ and *Pkd1* ^f/f^:HoxB7-Cre mice. These target proteins were significantly repressed in collecting duct-specific cyst lining epithelial cells (green), but not in normal tubules. Cy, cyst. Scale bar indicates 20 μm. (**C**) Five hours after scratching, mIMCD cells transfected with siRNAs for *Wasf2*, *Dock1*, or *Itga4* formed fewer lamellipodia and reduced expression of actin cytoskeleton proteins on lamellipodia at the leading edge compared with either control siRNA- or negative control mimic-transfected cells. Scale bars represent 25 μm (in the smaller square) and 75 μm. *P < 0.05; **P < 0.01; ***P < 0.001.

**Figure 5:**
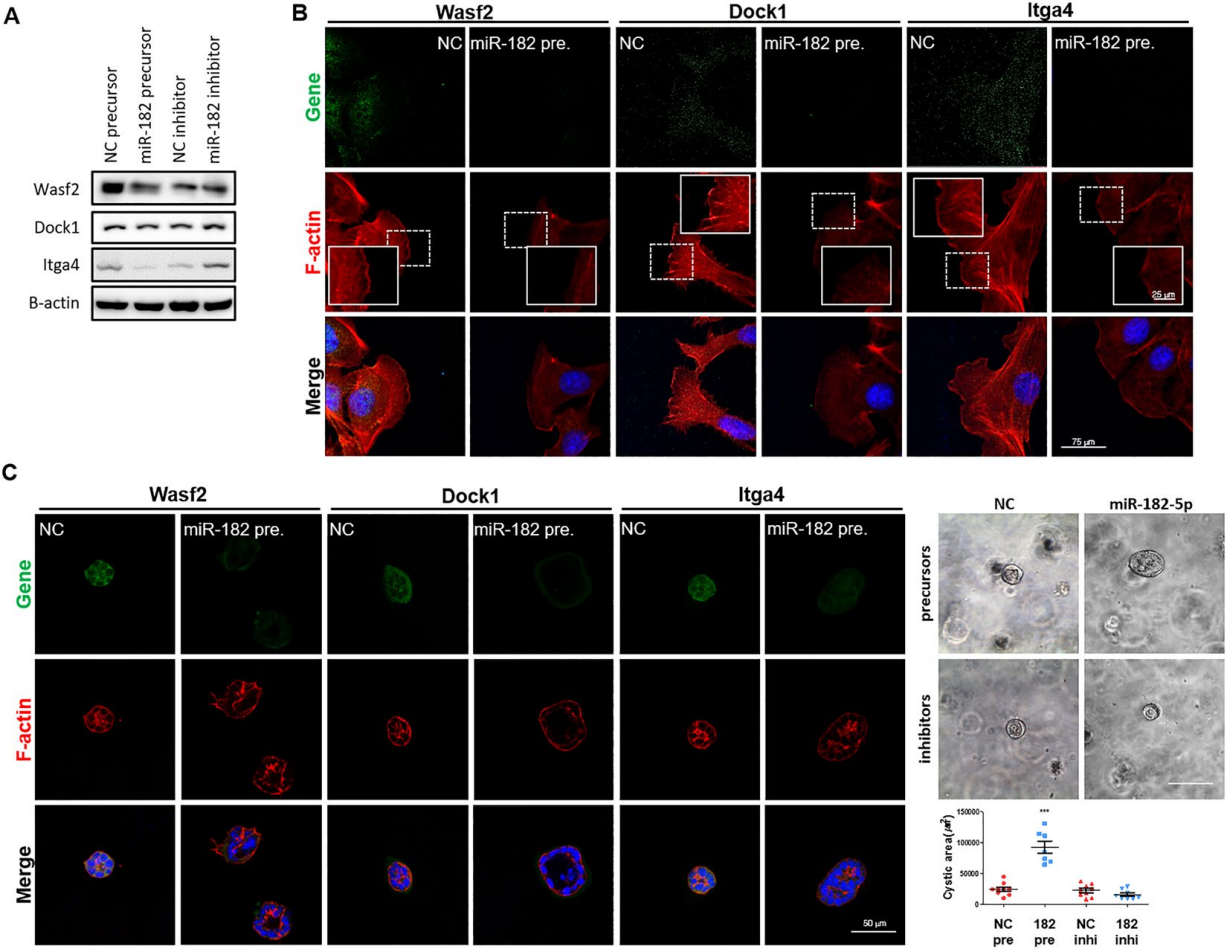
Role of miR-182-5p in renal cystogenesis linked to defects in the actin cytoskeleton. (**A**) Altered protein expression levels observed upon transfection of mIMCD cells with miR-182-5p mimic or miR-182-5p inhibitor were confirmed by western blot analysis. β-Actin was used as an internal control. Each experiment was performed in triplicate. (**B**) Pictures were taken five hours after scratching. Ectopic expression of miR-182-5p prevented the reorganization of actin filaments in mIMCD cells. Scale bars represent 25 μm (in the smaller square), and 75 μm. (**C**) miR-182-5p precursors or inhibitors (20 nM) were transfected into mIMCD cells, and then cells were observed in 3D culture conditions. The cysts in miR-182-5p precursor-treated cells were bigger than cysts in NC treated cells. miR-182-5p inhibitor-transfected mIMCD cells showed reduced of cyst lumen size. Immunostaining with Wasf2, Dock1, and Itga4 (green); F-actin (red); DAPI (blue). ***P < 0.001.

The overexpression of PC1 induces cytoskeletal rearrangement, cell migration, and tubule formation^32^. To evaluate the relevance of this protein to the actin cytoskeleton in ADPKD cystogenesis, 3-dimensional (3D) culture and F-actin staining were performed after knockdown of either *Pkd1* or *Pkd2* in mouse inner medullary collecting duct (mIMCD) cells. Prior to staining, mRNA and protein levels of *Pkd1*, *Pkd2*, and miR-182-5p target genes were determined in recovered mIMCD spheroids (Supplementary Fig. S7). Knockdown of either *Pkd1* or *Pkd2* in mIMCD cells resulted in remarkable defects of F-actin structure and accelerated cyst growth in 3D culture (Fig. 4A). In this study, we utilized two collecting duct-specific polycystin-1 knockout mouse models, *Pkd1* ^f/f^:HoxB7-cre and *Pkd1* ^f/f^:Aqp2-cre; two different promoters were used to drive Cre recombinase expression in the transgenic mice. Although *Pkd1* ^f/f^:HoxB7-cre mice and *Pkd1* ^f/f^:Aqp2-cre mice have similar PKD phenotypes, *Pkd1* ^f/f^:Aqp2-cre mice tend to survive longer than *Pkd1* ^f/f^:HoxB7-cre mice^33^. Upon staining these cells with both rhodamine-labeled phalloidin and marker genes related to the actin cytoskeleton in wild-type (*Pkd1* ^f/f^) and *Pkd1*-deficient (*Pkd1* ^f/f^:Aqp2-cre and *Pkd1* ^f/f^:HoxB7-cre) mouse kidneys, strong F-actin signals were observed in the apical membrane with weak signals outlining the tubules in WT kidneys. However, cyst-lining epithelial cells in *Pkd1*-deficient kidneys showed reduced and disorganized actin cytoskeletons with significantly decreased levels of marker genes such as *Wasf2*, *Dock1*, and *Itga4* (Fig. 4B and Supplementary Fig. S8). To investigate whether actin filament polymerization was involved in cyst development, actin structures were investigated after a wound healing assay. Five hours after scratching, cells deficient in *Wasf2*, *Dock1*, or *Itga4* did not produce organized actin cytoskeletons like those observed in the control siRNA-transfected cells (Fig. 4C). In addition, knocking down *Wasf2*, *Dock1*, or *Itga4* promoted cyst growth with actin cytoskeleton defects in 3D culture conditions (Fig. 4D). Moreover, western blotting analysis revealed that miR-182-5p negatively regulated the target genes *Wasf2*, *Dock1*, and *Itga4* (Fig. 5A). Intriguingly, we found that overexpressing miR-182-5p inhibits actin cytoskeleton organization, particularly lamellipodium formation, by repressing marker genes related with the actin cytoskeleton, ultimately affecting cyst development *in vitro* (Fig. 5B and C). Finally, overexpressing miR-182-5p increased cyst growth, while inhibiting miR-182-5p inhibited cyst formation (Fig. 5C). These data suggest that miR-182-5p may play a critical role as a novel inducer of cystogenesis by regulating the actin cytoskeleton.

### Inhibiting miR-182-5p attenuates cyst growth in a *Pkd1* conditional knockout mouse model

**Figure 6:**
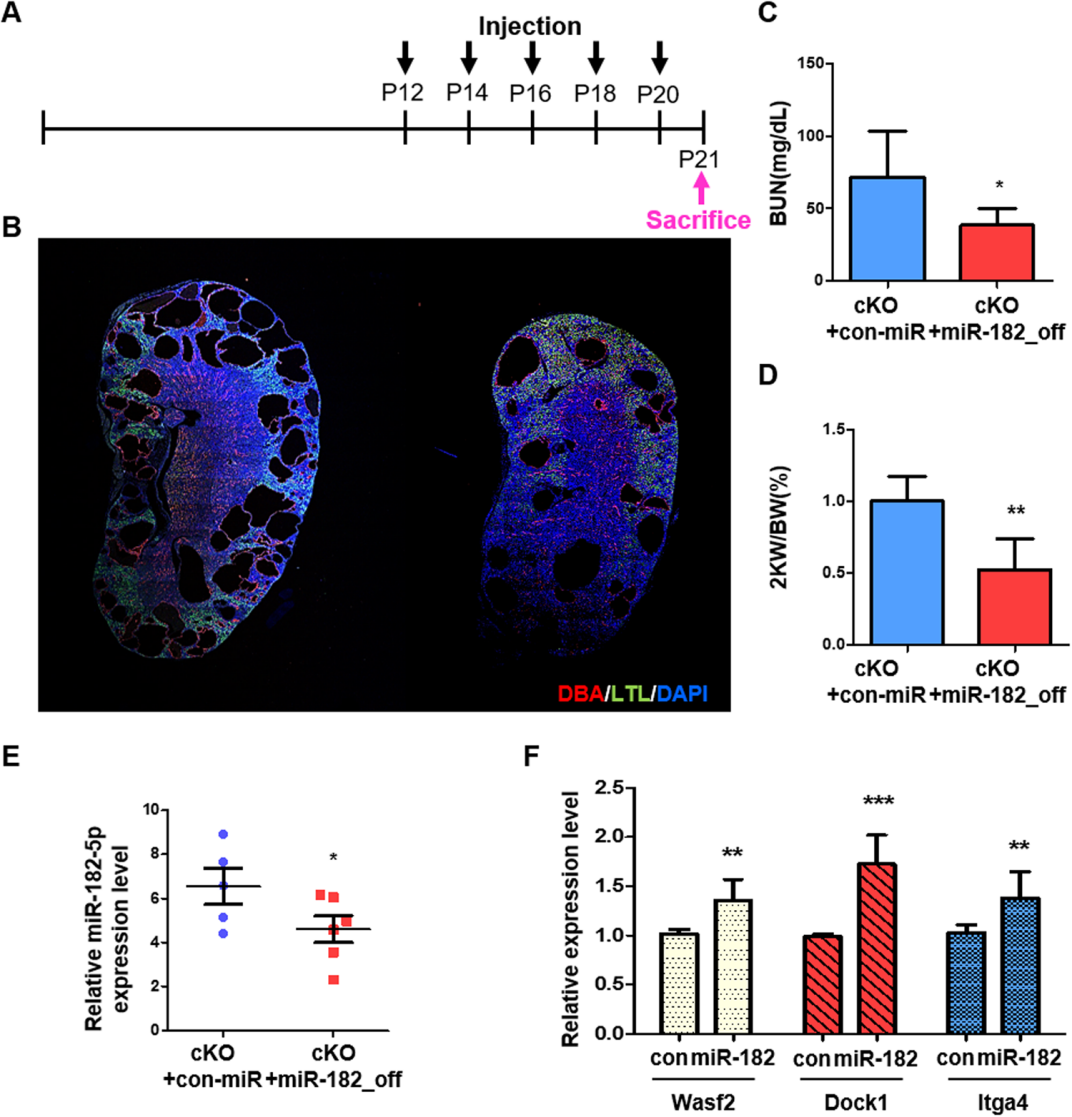
The effect of miR-182-5p inhibition in attenuating cyst growth *in vivo*. (**A**) The schedule for the injection study. (**B**) Immunofluorescence staining of kidney sections from control lentivirus- (left) or inhibitory miR-182-5p lentivirus- (right) injected *Pkd1* ^f/f^:Aqp2-Cre mice. Red indicates collecting duct, green indicates proximal tubule, and blue indicates the nucleus. (**C**) Serum BUN levels, (**D**) kidney weight-to-body weight ratio, (**E**) expression of miR-182-5p and (**F**) expression of *Wasf2, Dock1*, and *Itga4*. n = 5, control lentivirus- and n = 6, inhibitory miR-182-5p lentivirus-injected mice, respectively *P < 0.05; **P < 0.01; ***P < 0.001.

To evaluate the therapeutic effect of miR-182-5p inhibition, a lentiviral particle-mediated miRNA inhibition system was introduced. The *Pkd1* ^f/f^:Aqp2-cre mouse model was selected for the injection study because *Pkd1* ^f/f^:HoxB7-cre mice live for only 14–23 days. A control lentivirus or inhibitory miR-182-5p lentiviral particles (final conc.: 4 × 10^7^ IU) were injected intraperitoneally five times on alternate days. Mice were then sacrificed the day after the final injection (Fig. 6A). Kidney weight-to-body weight ratios, BUN levels, and miR-182-5p expression in kidney tissues were reduced upon treatment with the inhibitory miR-182-5p lentivirus compared with those in con-lentivirus-treated *Pkd1* ^f/f^:Aqp2-cre mice (Fig. 6B–E). Furthermore, we observed that the expression patterns of the target genes of miR-182-5p, *Wasf2*, *Dock1*, and *Itga4*, were restored in mice treated with inhibitory miR-182-5p lentivirus (Fig. 6F). Collectively, these results indicated that inhibiting miR-182-5p was effective for inhibiting cyst growth and restoring renal function.

## Discussion

In the current study, we described parallel integrated analysis of miRNA-seq and RNA-seq data and validated the expression, direct interaction, and cystogenesis-related functions of key miRNAs and their targets. In particular, we found commonly dysregulated pathways by comparing *Pkd1*- and *Pkd2*-deficient mouse models. These models had overlapping, differentially expressed miRNAs and signaling pathways related with cystogenesis, suggesting that these miRNAs play crucial roles in cyst formation and expansion in ADPKD patients with either *PKD1* or *PKD2* mutations. Although both mouse models have common key miRNAs, these miRNAs are considered to target different mRNAs in *Pkd1* and *Pkd2*-deficient mice (Supplementary Fig. S9). Indeed, our data showed that miR-182-5p was associated with cystogenesis by regulating the actin cytoskeleton signaling pathway in both mouse models. However, miR-182-5p directly targets and regulates *Wasf2*, *Dock1*, and *Itga4* in *Pkd1*-deficient mice, but not in *Pkd2*-deficient mice (Supplementary Fig. S10). This difference may be related to their phenotypic differences: even though these two models showed similar cyst progression and timing of renal failure, *Pkd2*-deficient mice remained alive longer than *Pkd1*-deficient mice, for maximum of about 20 days. This suggests that these mouse models share common molecular alterations in early stage cystogenesis but they have specific molecular changes in late stages, at least after P7. Therefore, our data showed that defects in *Pkd1* or *Pkd2* share some, but not all, molecular changes and signaling pathways. Moreover, the parallel analysis between time-dependent phenotypic characterization and miRNA-mRNA profiling allowed us to select and validate more precise targets involved in ADPKD cyst progression.

Recently, there have been several miRNA profiling studies in ADPKD rodent models, using microarrays. Pandey *et al*. performed global gene-expression profiling by microarray in *Pkd1* ^−/−^ embryonic kidneys at P14.5 and 17.5 and predicted miRNAs through computational approaches^34^. They confirmed nine candidate miRNAs, including miR-182 and miR-96, by qPCR and suggested that several miRNAs may be associated with ADPKD-related signaling pathways. Dweep *et al*. identified eight significantly upregulated miRNAs in diseased kidneys of PKD/Mhm rats by a parallel microarray-based approach^35^. A major advantage of unbiased next-generation sequencing-based approaches such as small RNA-seq and RNA-seq is that they provide genome-wide and quantitative information, whereas microarray-based approaches do not. Our results are partially in agreement with two previous studies but have different candidate miRNAs and targeting signaling pathways. Although it is well-known that increased proliferation by pathways including ERK/MAPK is required for cyst formation, the precise mechanism of cyst formation and enlargement in ADPKD has not yet been described. This indicates that systemic analysis is necessary for uncovering novel relevant pathways. From integrated miRNA-seq and RNA-seq analysis, we observed that *Pkd1* inactivation changes the expression of several key miRNAs involved in previously reported signaling pathways such as ERK/MAPK and Akt/mTOR signaling, as well a novel pathway, actin cytoskeleton signaling. In addition, our data showed that upregulation of miR-21a-5p in cyst-lining epithelial cells stimulates cyst growth in *Pkd1*-deficient mice, consistent with a previous study^23^.

Intriguingly, we provided evidence of a direct link between the actin cytoskeleton and cystogenesis in ADPKD. In a previous study, polycystins were found to be responsible for cell migration, polarity, and tissue morphogenesis^36^. PC1 regulates the actin cytoskeleton and cell migration mediated by the phosphatidylinositol 3-kinase (PI3-K) pathway in MDCK cells^32^. The PI3-K pathway was significantly enriched in diseased kidneys of *Pkd1*-deficient mice, suggesting that the downregulation of PI3K genes such as *Pik3r1* and *Pik3r3* may be associated with actin cytoskeleton defects and reduced migration capacity in cystic epithelial cells. In addition, PC1 is involved in the actin cytoskeleton by interacting with the Pacsin/WASP-2/Arp2/3 complex, which contributes to microfilament nucleation at the leading edge^18^. However, these two studies did not show direct evidence linking the actin cytoskeleton with cyst development. From our results, we identified that key proteins involved in the actin cytoskeleton were regulated by several miRNAs, including miR-182-5p, that control cyst progression. Wasf2, also known as Wave2, is a well-known key component of the actin regulatory machinery and mediates actin cytoskeletal organization by cooperating with the Arp2/3 complex to promote the formation of actin filaments^37^. We identified, *Wasf2* as a novel direct target of miR-182-5p. Inhibiting *Wasf2* expression accelerated cyst growth, dysregulated actin structure, and decreased cell migration. We also found that *Dock1* is a novel direct target of miR-182-5p. Dock1 is a guanine nucleotide exchange factor that activates the Rac1/WAVE-complex/Arp2/3-complex, resulting in the formation of lamellipodial protrusions^38^. We observed that *Dock1* repression also stimulated cyst development via alterations of the actin structure in mIMCD 3D cultures. Taken together, the results suggest that defects in the actin cytoskeleton and progression of cyst formation are tightly linked. Furthermore, actin dynamics seem to be linked to establishment of cell polarity and formation of apical junctions^39^. Defects in cell/matrix interaction is a suggested causative mechanism of cystogenesis in ADPKD, but these remain poorly understood. Integrins are the main cell surface receptors for proteins within the extracellular matrix (ECM)^40^. They contribute to cell movement and mediate adhesion junctions, which are essential for tissue integrity^41^. Integrin alpha 4 (Itga4), which we identified as a novel target of miR-182-5p, may play a crucial role in migratory events and we showed that it can affect cyst growth rate *in vitro*.

Considering our observations in this study, miR-182-5p should be a potential therapeutic target. We also observed that miR-182-5p inhibition by systemic injection of lentiviral particle-mediated inhibitory miRNA attenuated cyst progression *in vivo*. We confirmed that the expression of *Wasf2*, *Dock1*, and *Itga4* were restored in the miR-182-5p lentivirus-injected *Pkd1* ^f/f^:*Aqp2*-cre mice. However, miR-182-5p inhibition could not slow cyst growth in *Pkd2* ^f/f^:Aqp2-cre mice because *Wasf2*, *Dock1*, and *Itga4* play significant roles in *Pkd1* but not in *Pkd2* conditional KO mice. The miRNA and its target genes, other than miR-182-5p, may play more important regulatory functions in *Pkd2* conditional KO mice. Overall, we present strong evidence that miR-182-5p upregulation is *Pkd1*-dependently associated with defects in the actin cytoskeletal pathway, which can cause symptoms of PKD.

In conclusion, we applied a NGS-based methodology to identify several key miRNAs that are linked to *Pkd1* or *Pkd2* expression using two ADPKD mouse models, namely, conditional *Pkd1* or *Pkd2*-deficient mice. The miRNA-mRNA integrated data for cyst progression at three time-points revealed genome-wide correlations between molecular changes and disease phenotype. Additionally, we provided evidence of a direct link between the actin cytoskeleton and cystogenesis in ADPKD. Therefore, we suggest that these key miRNAs and their possible potential targets play a central role in cystogenesis in ADPKD.

## Methods

### Animal Models

Both the floxed *Pkd1* (*Pkd1* ^flox/flox^) containing loxP sites inserted in the genomic DNA flanking exon 2–5 of the *Pkd1* gene^26^ and floxed *Pkd2* (*Pkd2* ^flox/flox^) containing loxP sites flanking exon 3 and 4 of *Pkd2* gene mice^42^ were obtained from Dr. Stefan Somlo at Yale University. HoxB7-Cre transgenic mice, which specifically drives cre expression in the kidney collecting duct, was obtained from Minho Shong at Chungnam National University. To characterize the phenotype resulting from *Pkd1* or *Pkd2* inactivation in the collecting duct, *Pkd1* ^f/+^:HoxB7-Cre and *Pkd1* ^f/f^ or *Pkd2* ^f/+^:HoxB7-Cre and *Pkd2* ^f/f^ mice were mated to generate a conditional knockout mouse model. Age-matched littermates with *Pkd1* ^f/f^ or *Pkd2* ^f/f^ genotypes were used as controls. Genotyping was performed using previously described protocols^26^. Tail DNA was PCR-amplified for *Pkd1* ^f/f^ alleles using the forward primer 5′-CCGCTGTGTCTCAGTGCCTG-3′ and reverse primer 5′-CAAGAGGGCTTTTCTTGCTG-3′; for *Pkd2* ^f/f^ alleles using the forward primer 5′-GGGTGCTGAAGAGATGGTTC-3′ and reverse primer 5′-TCCACAAAAGCTGCAATGAA-3′. HoxB7-cre transgene expression was confirmed using the forward primer 5′-GCGGTCTGGCAGTAAAAACTATC-3′ and reverse primer 5′-GTGAAACAGCATTGCTGTCACTT-3′. All experimental procedures were performed in accordance with relevant guidelines and regulations on laboratory animal care. All experimental protocols were reviewed and approved by the IACUC at Sookmyung Women’s University.

### Cell culture and transfection

mIMCD cells were cultured in DMEM/F12 (Welgene, Korea) media with 10% (v/v) fetal bovine serum (FBS), and 1% penicillin-streptomycin (Welgene, Korea) in a humidified 5% CO_2_ atmosphere at 37 °C. Small interfering RNA (siRNA) targeting mouse *Wasf2*, *Dock1*, or *Itga4* (Bioneer, Korea) genes were transfected into mIMCD cells at 20 nM using Lipofectamine RNAiMAX reagent (Invitrogen, Carlsbad, CA, USA) according to the manufacturer’s instructions for 24 to 48 h. miRNA mimics and inhibitors for mmu-miR-182-5p (Ambion) were reverse-transfected into mIMCD cells at 10 nM using siPORT NeoFX transfection reagent (Ambion) for 24 to 48 h. The control experiments were transfected with scramble miRNA (Pre-miR miRNA Precursor and miR-Vana miRNA inhibitor Molecules, Negative Control #1, Ambion).

Three-dimensional (3D) cell culture was performed according to previously reported protocol^43^. Briefly, suspended mIMCD cells (1 × 10^4^ cells/well) were mixed with Matrigel at a 1:1 ratio and plated in 8-well chamber slides. Cells in Matrigel were grown in a humidified 5% CO_2_ atmosphere at 37 °C for 5 days. After 5 days in 3D culture, phase contrast images of individual spheroids were obtained by a light microscope (IX70, Olympus) and cyst area was measured using ISP capture software (Olympus).

### Immunostaining, immunofluorescence microscopy, and western blots

Kidneys were fixed in 4% paraformaldehyde overnight, then paraffin-embedded. 5 μm-width sections were deparaffinized in three changes of xylene, followed by rehydration in an ethanol series. Sections were heated in 0.01 M citric acid (pH 6.0) for antigen retrieval and blocked with blocking solution for 1 h at room temperature followed by overnight incubation with the appropriate primary antibody at 4 °C. Primary antibodies used for immunostaining were: Rhodamine-labeled DBA (RL-1032, Vector laboratory), Fluorescein-labeled LTL (FL-1321, Vector laboratory), Wasf2 (H-110, Santa Cruz Biotechnology, sc-33548), Dock1 (H-4, Santa Cruz Biotechnology, sc-13163), and Itga4 (H-210, Santa Cruz Biotechnology, sc-14008).

For phalloidin staining of the actin cytoskeleton in kidney tissues, tissues were fixed in 4% paraformaldehyde in PBS and embedded in OCT. Cryosections were blocked with 1% BSA in PBS for 1 h at room temperature and incubated with rhodamine-labeled phalloidin (Invitrogen, Catalog #R415) diluted 1:500 overnight at 4 °C. Slides were mounted using mounting medium with DAPI (Vector laboratory).

For immunocytochemistry, cultured cells were fixed in 4% paraformaldehyde for 10 min at room temperature, and then washed three times with PBS. Cells were permeabilized with 0.2% Triton X-100 containing 5% v/v bovine serum albumin, and incubated with the appropriate primary antibody at 4 °C overnight. Images were obtained with a confocal laser scanning microscope (LSM-700, Carl Zeiss).

For western blot analysis, equal amounts of protein were analyzed in duplicate by SDS-PAGE and electro-transferred to a polyvinylidene fluoride (PVDF) membrane (ATTO, Japan). Primary antibodies were diluted at 1:200~1:1000 in 1% skim milk in PBST at 4 °C overnight and secondary antibodies were incubated for 1 h at room temperature. Anti-β-actin (Bethyl Laboratories, Montgomery, TX, USA) was used as a loading control. Immunoreactive proteins were detected by horseradish peroxidase–conjugated secondary antibodies and enhanced using chemiluminescence (ECL) reagents (GE Healthcare Life Sciences, Piscataway, NJ).

### Cystic index measurement and renal function test

The tubular cyst formation areas were quantified in whole kidney sections of *Pkd1* ^f/f^:HoxB7-Cre and *Pkd2* ^f/f^:HoxB7-Cre mice over time. Two sections from the mid-sagittal region of each kidney were analyzed on P1, P3, and P7 for each mouse model (n = 3). Whole kidney images stained with tubule markers were obtained using the scan slide module in a confocal laser scanning microscope (LSM-700, Carl Zeiss). Total kidney area and cystic area with DBA signal were measured using the tools provided by LSM-700. The total kidney area was divided into four parts according to their sizes: non-cystic area, dilated tubules (<1 × 10^5^ μm^2^), small cysts (1 × 10^5^–10^6^ μm^2^), and large cysts (>1 × 10^6^ μm^2^). Cyst index = (total cystic area/total kidney area) × 100 (%).

To investigate renal function, serum was isolated from blood samples using Vacutainers (BD Biosciences) and blood urea nitrogen (BUN) analysis was performed by Dr. Chulho Lee’s laboratory using a Hitachi 7150 Clinical Analyzer (KRIBB, Daejeon, Korea).

### Small RNA and mRNA sequencing, alignment, and normalization

Total RNA was isolated using TRIzol according to the manufacturer’s protocol (Invitrogen Life Technologies). To estimate expression levels and to determine alternatively spliced transcripts, the RNA-Seq reads were mapped to the mouse genome (*Mus musculus*, version mm10) downloaded from the UCSC website (http://genome.uscs.edu) using TopHat v2.1.0^44^, which is capable of reporting split-read alignments across splice junctions. Read counts of representative genes and their isoforms were measured by HTSeq^45^ with default options using RefSeq annotation as a reference. Read counts under ten counts in at least three samples were filtered out before normalization, and normalization was performed using the TMM method^46^. Statistical significance of the transcript expression data was determined with criteria (two-fold change and false discovery rate (FDR < 0.05)) from the edgeR^30^ Deseq2^31^ implemented in the Bioconductor package. Differentially expressed genes (DEGs) were identified by pairwise comparisons between WT and KO groups at each time point. The DEG sets were collected for all three time points by combining the lists obtained by edgeR and Deseq2. Hierarchical clustering for DEGs was performed using complete linkage and Euclidean distance as a measure of similarity. All statistical analysis and visualization of differentially expressed genes was conducted using R 3.2.3 (www.r-project.org).

For miRNA-Seq analysis, raw reads were trimmed by removing the 3′ adaptor sequence and retaining more than 17 bp of the read sequence. For read mapping of miRNA, trimmed reads were aligned against the mouse genome or aligned to the known mature & hairpin miRNAs (miRBase version 21^47^, June, 2014) by the miRdeep2 program^48^. Raw read counts for miRNA were filtered and normalized by the same process of RNA-Seq data analysis. Differentially expressed miRNAs (DEmiR) were classified as genes having a 2 fold-change and P-value < 0.05. The DEmiR sets were determined for all three time points by combining the lists obtained by edgeR and Deseq2.

### Integration of miRNA-mRNA data

We performed mRNA-miRNA data integration for matched samples in each data set. First, the DEG set and miRNA set were used to calculate Spearman’s rank correlation between miRNA and mRNA expression in all data sets. This approach assumed that the expression of a given miRNA is negatively correlated with the mRNA expression of its targets. For miRNAs with the potential to regulate mRNA, a correlation test was performed in R to determine if the miRNA was statistically significant with a P-value < 0.05 and a negative correlation coefficient. Then we retained putative miRNA-target mRNA pairs with a relationship using miRwalk v2.0^49^ (http://zmf.umm.uni-heidelberg.de/apps/zmf/mirwalk2/), a database that supplies the largest available collection of predicted and experimentally verified miRNA-target interactions.

### Pathway and networking analysis

Putative target gene sets of differentially expressed miRNAs were evaluated by QIAGEN’s Ingenuity Pathway Analysis (IPA, QIAGEN Redwood City, www.qiagen.com/ingenuity) for biological functions and canonical pathways or networks. Then, we identified canonical pathways associated with ADPKD phenotype by identifying genes correlated with miRNAs. The network analysis tool Cytoscape v3.3 (http://cytoscape.org/) was used to find the putative target genes for the predicted miRNAs in canonical pathways. Specially, gene-gene interactions were obtained from Pathway Studio^50^ to identify highly connected modules within a canonical pathway.

### Quantitative real-time RT-PCR

To detect mRNA expression, total RNA (2 μg) was reverse-transcribed using M-MLV Reverse Transcriptase (Promega), 100 nM oligo-dT, 1 mM dNTP mixture, and RNase inhibitor. Mouse 18S rRNA, detected with the forward primer 5′-GTAACCCGTTGAACCCCATT-3′ and reverse 5′-CCATCCAATCGGTAGTAGCG-3′, was used as an endogenous control. Quantitative real-time PCR was performed using the real-time SensiMixPlus SYBR kit as described in the manufacturer’s instructions (Quantance, London, UK).

To detect miRNA expression, cDNA was prepared from total RNA (500 ng) using a miScript II RT kit (Qiagen) according to the manufacturer’s protocol. Real-time PCR quantification of mature miRNA was performed using target-specific miScript Primer Assays (Qiagen) and the miScript SYBR Green PCR Kit (Qiagen) according to the manufacturer’s protocol.

### 3′UTR reporter analysis

The 3′UTR of mmu-miR-182-5p candidate target mRNAs were amplified by PCR using PrimeSTAR GXL DNA polymerase (Takara Bio Inc., Japan) and cloned into the *Xho*I and *Not*I site of a psiCHECK-2 vector (Promega) using an In-Fusion HD Cloning kit (Takara Bio Inc., Japan). The seed sequences of miR-182-5p were mutated using a PCR-based approach. For transfection, mIMCD cells (2 × 10^5^) were plated into 6-well plates and 1.5 μg of luciferase constructs and 15 nM of negative control mimics or mmu-miR-182-5p mimics (Ambion) were co-transfected into the cells one day after seeding using Lipofectamine 2000 (Invitrogen) according to the manufacturer’s instruction. Cells were collected after 36 h for experiments and luciferase activity was measured by a Dual Luciferase Assay system (Promega). All transfection experiments were performed in triplicate and at least three times.

### Section microRNA ***in situ*** hybridization

Kidneys were dissected at postnatal day 7 and subsequently fixed in 4% paraformaldehyde (PFA) overnight. After fixation, tissues were embedded in paraffin and sectioned at a thickness of 7 μm. Sections were incubated at 60 °C for deparaffinization using Histoclear II (National Diagnostics), re-hydrated through a graded series of alcohol washes and 1X DEPC-PBS. The specimens were treated with 10 μg/ml proteinase K for 5 min at 37 °C. Sections were incubated in pre-hybridization buffer (50% formamide, 5× SSC, 0.1% Tween-20, 50 ug/ml heparin, 500 ug/ml yeast tRNA) at 53 °C for 1 hr. Hybridization was conducted at 53 °C for overnight using miR-182 locked nucleic acid (LNA) modified probe (Exiqon). Sites of hybridization were detected using alkaline phosphatase-conjugated DIG antibody (Roch) at a 1:1,000 dilutions, RT for overnight, followed by NBT/BCIP AP substrate color development (Roche).

### Statistical analysis

Each experiment was repeated at least three times throughout the study. Data were reported as means ± SD. Statistical significance was determined by Student’s *t*-tests when only two groups were compared (GraphPad Software, San Diego, CA). *P* < 0.05 was considered significant.

### Accession codes

mRNA and Small RNA sequencing data have been deposited in the NCBI’s Gene Expression Omnibus (GEO) Database under the accession code GSE86509 and in the Korean Bioinformation Center (KOBIC)’s database under the accession codes KBRS20160928_0000001 ~ KBRS20160928_0000072.

## Electronic supplementary material

Suppplementary figures

Suppplementary Table S1

Suppplementary Table S2

Suppplementary Table S3

Suppplementary Table S4

Suppplementary Table S5

Suppplementary Table S6
