# Supplementary material for: Profiling of miRNAs and target genes related to cystogenesis in ADPKD mouse models
Source: Sci Rep. 2017 Oct 26;7:14151. doi: 10.1038/s41598-017-14083-8 (PMC5658336; doi:10.1038/s41598-017-14083-8)
Supplement: Supplementary file 1 — Suppplementary figures [file 41598_2017_14083_MOESM1_ESM.pdf]

Profiling of miRNAs and target genes related to cystogenesis in ADPKD mouse models

Yu Mi Woo[1#], Do Yeon Kim[1#], Nam Jin Koo[2#], Yong-Min Kim[2], Sunyoung Lee[1], Je Yeong Ko[1], Yubin Shin[1], Bo Hye Kim[1], Hyowon Mun[1], Seonju Choi[1], Eun Ji Lee[1], Jeong-Oh Shin[3], Eun Young Park[1], Jinwoong Bok[4], and Jong Hoon Park[1, *]

[1]Department of Biological Science, Sookmyung Women's University, Seoul, Republic of Korea

[2]Korean Bioinformation Center, Korea Institute of Bioscience and Biotechnology, Daejeon, 34141, Republic of Korea

[3]Department of Anatomy, Embryology Lab. College of Medicine Yonsei University, Seoul 120-752, Korea

[4]Departments of Anatomy and Otorhinolaryngology, and BK21 PLUS Project for Medical Science, Yonsei University College of Medicine, Seoul 03722, Republic of Korea

[#]These authors contribute equally to this work.

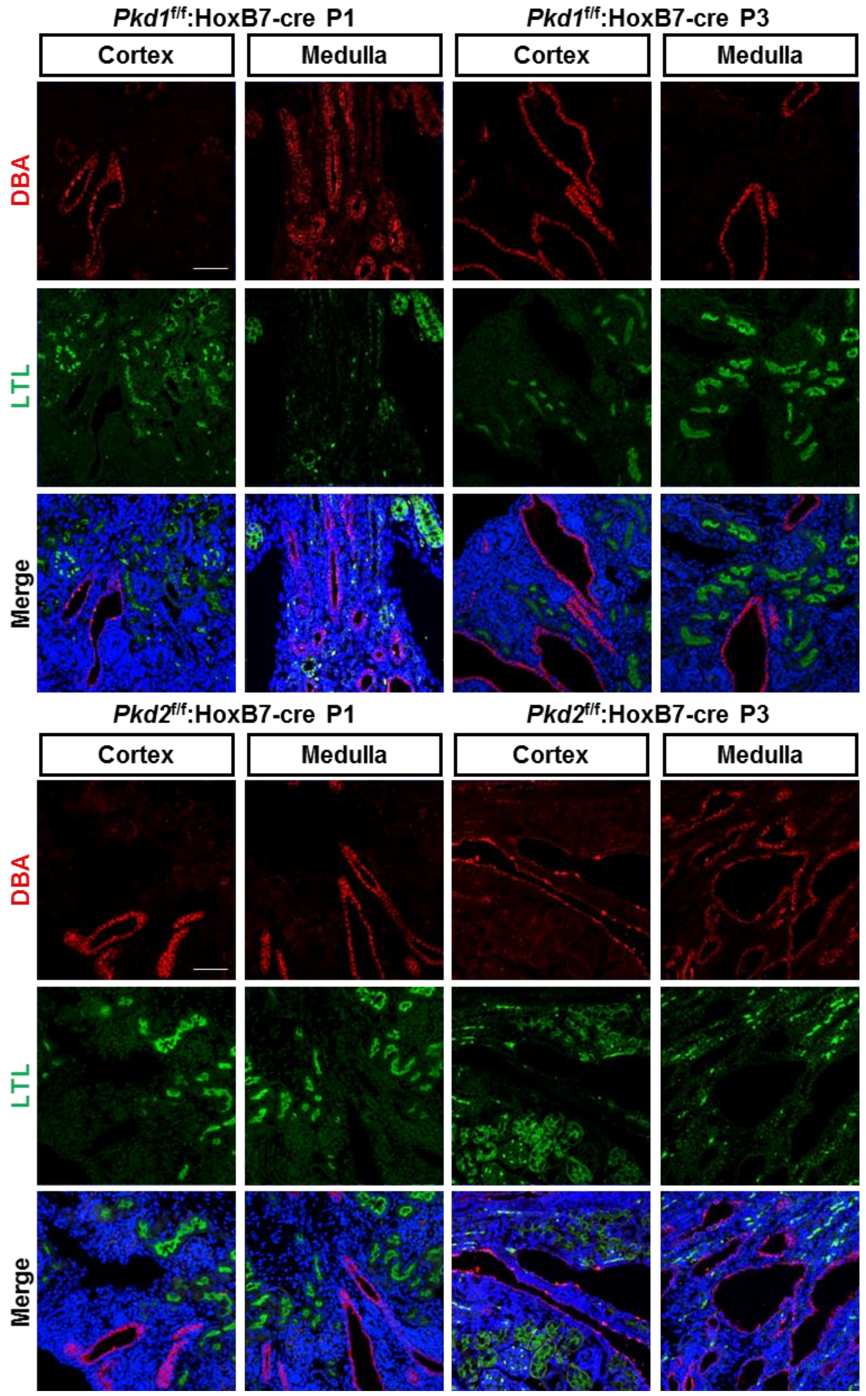

Supplementary Figure 1. Image of DBA-positive cysts in *Pkd1*$^{f/f}$:HoxB7-cre and *Pkd2*$^{f/f}$:HoxB7-cre at postnatal days P1 and P3. Magnification images of DBA-positive cysts in cortex and medulla regions from *Pkd1*$^{f/f}$:HoxB7-cre and *Pkd2*$^{f/f}$:HoxB7-cre mice at P1 and P3. Red indicates collecting duct, green indicates proximal tubule, and blue indicates the nucleus. Scale bars represent 50 μm.

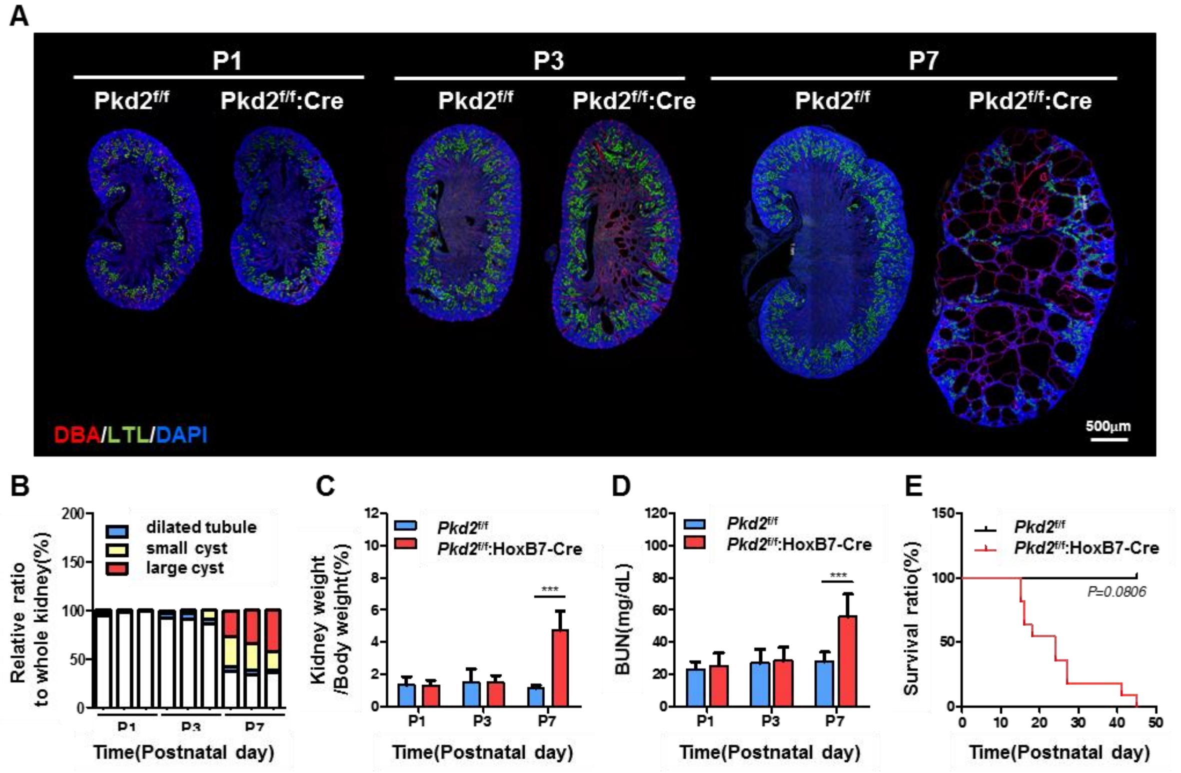

Supplementary Figure 2. Phenotype of *Pkd2* conditional knock out mouse in kidney collecting duct. (A) Kidney tissue from postnatal day 1, 3, and 7 of *Pkd2*$^{f/f}$ control mice, and *Pkd2*$^{f/f}$:HoxB7-Cre experimental mice detected by fluorescent immunohistochemistry. Red indicates collecting duct, green indicates proximal tubule, and blue indicates the nucleus. **(B)** The sum of single cyst areas to whole kidney area. **(C)** The ratio of kidney weight to body weight of *Pkd2*$^{f/f}$ and *Pkd2*$^{f/f}$:HoxB7-Cre mice. **(D)** Blood Urea Nitrogen level in *Pkd2*$^{f/f}$ and *Pkd2*$^{f/f}$:HoxB7-Cre mice. n(*Pkd2*$^{f/f}$)=10, n(*Pkd2*$^{f/f}$:HoxB7-Cre)=10. **(E)** Survival rate of *Pkd2*$^{f/f}$ and *Pkd2*$^{f/f}$:HoxB7-Cre mice. n=10. Data are presented as mean ±SD of three independent experiments in triplicate. ***, $P < 0.001$.

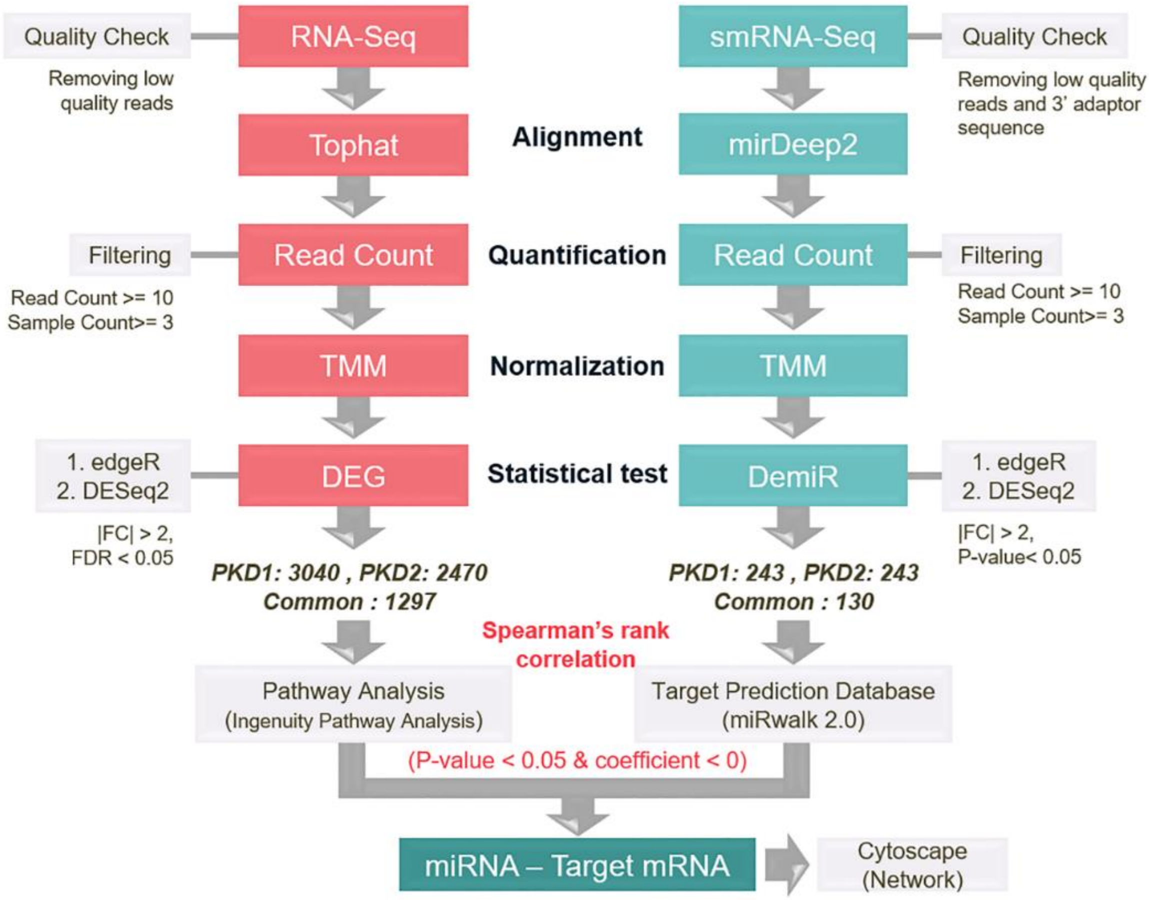

Supplementary Figure 3. Scheme of parallel analysis for RNA-seq and miRNA-seq. The kidney tissues of two mouse models at P1, P3, and P7 were analyzed by both miRNA-seq and RNA-seq. Expressions were measured by read counts based on known sequences. Significantly differentially expressed miRNAs (DEmiRs) and genes (DEGs) were identified using edgeR and Deseq2. Finally, 1,297 transcripts and 130 miRNAs were common in both mouse models. Selected DEGs and DEmiRs were tested by correlation analysis, and then pathway analysis was performed.

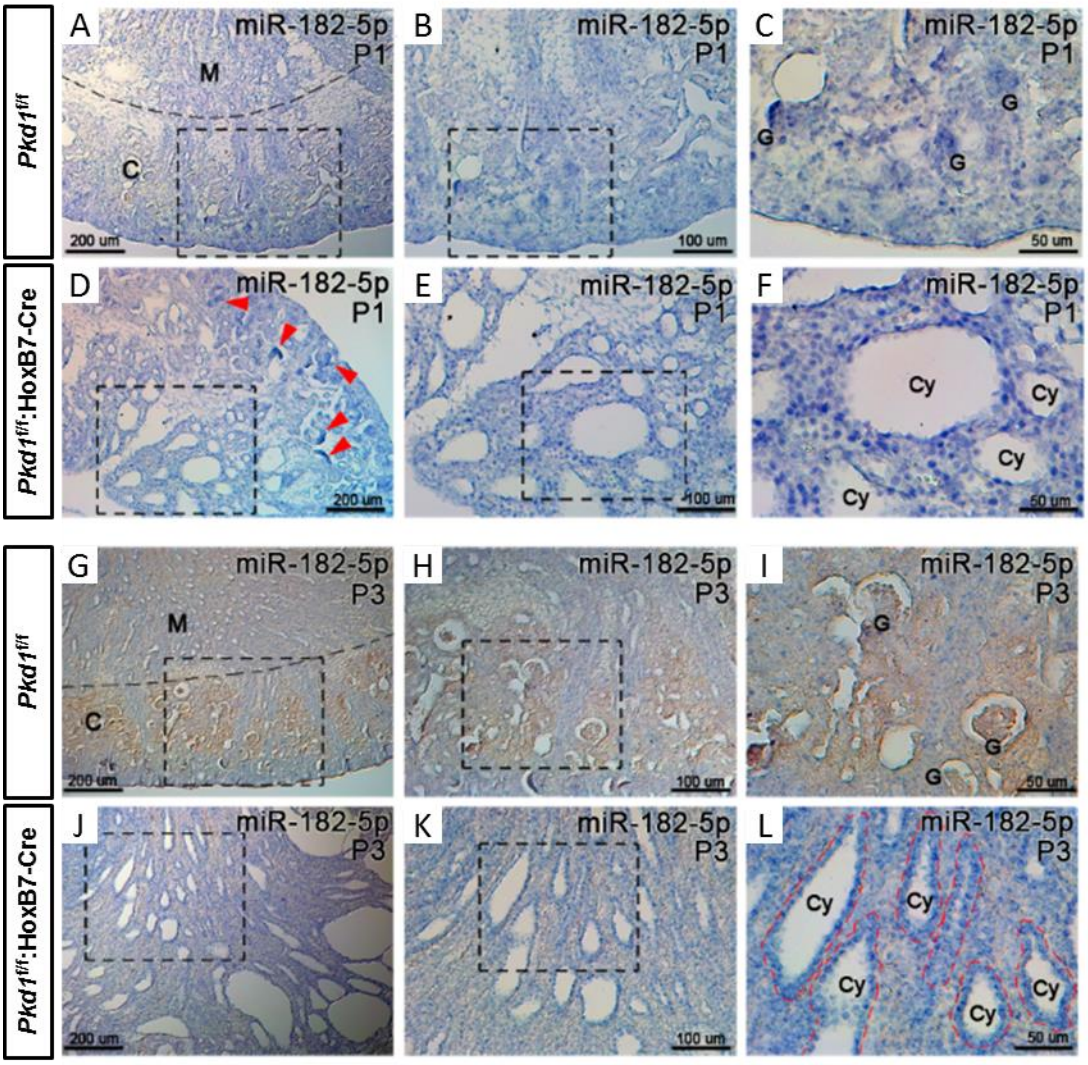

Supplementary Figure 4. Expression pattern of miR-182-5p during cystic kidney progression at postnatal days P1 and P3. (A-F) Representative section ISH for miR-182-5p from *Pkd1*$^{f/f}$ and *Pkd1*$^{f/f}$:HoxB7-Cre mice kidney demonstrates that miR-182-5p was detectable at P1. **(G-L)** ISH revealed that the overall region in P3 control kidney showed decreased expression compared to the P1 kidney, including the glomerulus and renal tubule. In the *Pkd1*$^{f/f}$:HoxB7-Cre mice kidney, miR-182-5p was strongly expressed in the cystic epithelial lining, but not in connective tissue near cysts at P3. M, medulla; C, cortex; G, glomerulus; Cy, cyst; red arrowhead, miR-182-5p-expressing glomerulus in the *Pkd1*$^{f/f}$:HoxB7-Cre mice kidney; black dotted box, high magnification region; red dotted line, cyst lining epithelial cells. Scale bars indicate 200 μm in A, D, G, and J; 100 μm in B, E, H, and K; 50 μm C, F, I, and L.

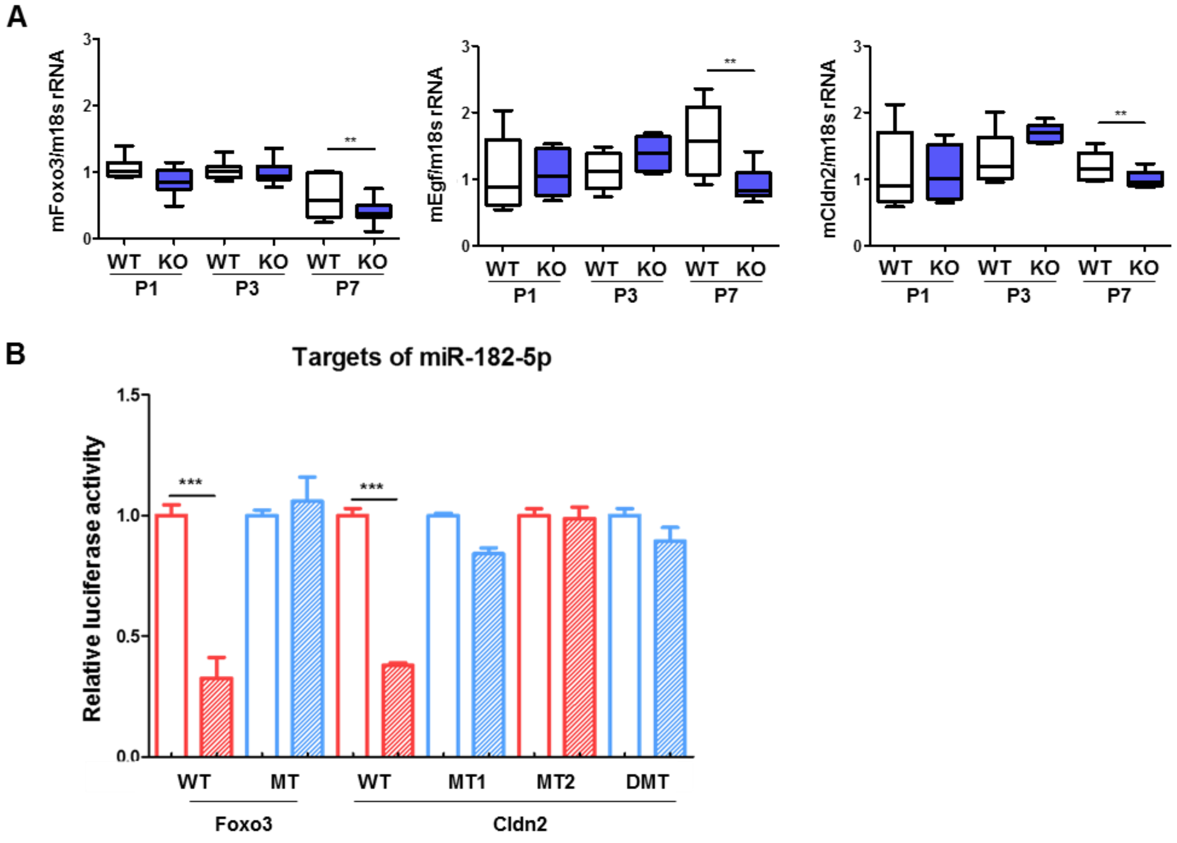

Supplementary Figure 5. Validation of candidate target mRNAs of miR-182-5p. (A) Candidate target mRNAs of miR-182-5p were confirmed in the kidneys of *Pkd1*$^{f/f}$:HoxB7-Cre at postnatal days P1, P3, and P7 using quantitative real-time RT-PCR. Target mRNAs included genes related with cystogenesis and the actin cytoskeleton (*Egf*, *Foxo3, Cldn2*). *18s rRNA* was used as an internal control for miRNA and mRNA. The experiment was performed in triplicate. n ≥ 3 for each time point. **(B)** Relative 3'UTR luciferase activity of *Foxo3* and *Cldn2* genes upon transfecting mIMCD cells with Negative Control mimic (NC mimic) or miR-182-5p mimic. Mutating the seed sequence of miR-182-5p induced rescued luciferase activity of the psiCHECK-2 vector. WT (wild type), MT (mutant type), DMT (double mutant type). Data are presented as mean ±SD of three independent experiments in triplicate. ***, *P* <0.001; **, *P* <0.01.

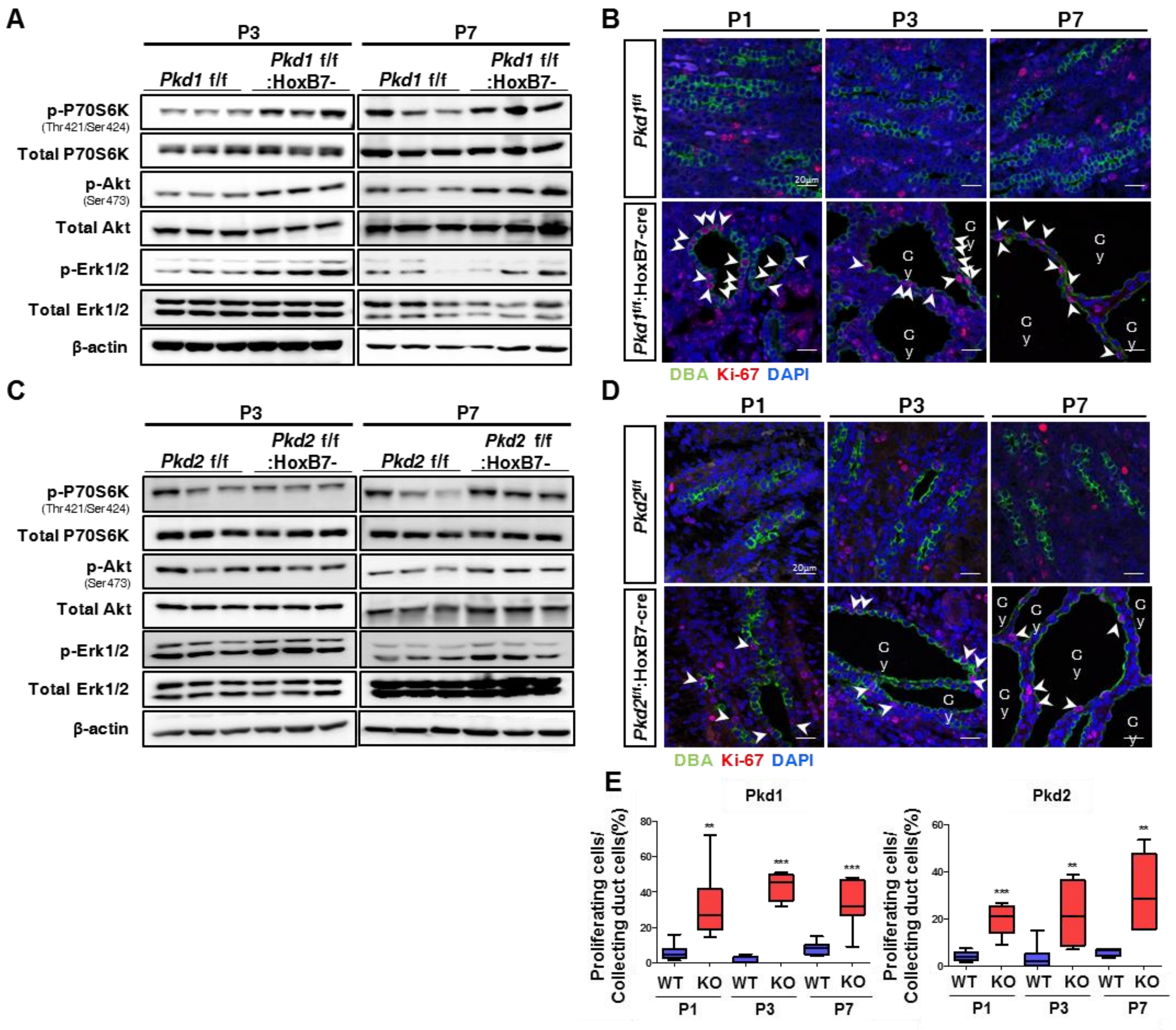

Supplementary Figure 6. Both *Pkd1* and *Pkd2* conditional knockout mouse models show increased ERK/MAPK and Akt/mTOR signaling and cell proliferation. (A, C) Proteins were obtained from *Pkd1*$^{f/f}$, *Pkd1*$^{f/f}$:HoxB7-Cre, *Pkd2*$^{f/f}$ and *Pkd2*$^{f/f}$:HoxB7-Cre mouse models at P3 and P7 time points. Molecules in ERK/MAPK and Akt/mTOR pathways were examined by western blotting. **(B, D)** Ki-67 stained cells were observed in *Pkd1*$^{f/f}$:HoxB7-Cre and *Pkd2*$^{f/f}$:HoxB7-Cre mouse models compared to control mice. **(E)** Two graphs showed percent of DBA positive cells per Ki-67 positive cells in *Pkd1*$^{f/f}$, *Pkd1*$^{f/f}$:HoxB7-Cre, *Pkd2*$^{f/f}$ and *Pkd2*$^{f/f}$:HoxB7-Cre mouse models at P1, P3, and P7. ***, P <0.001; **, P <0.01.

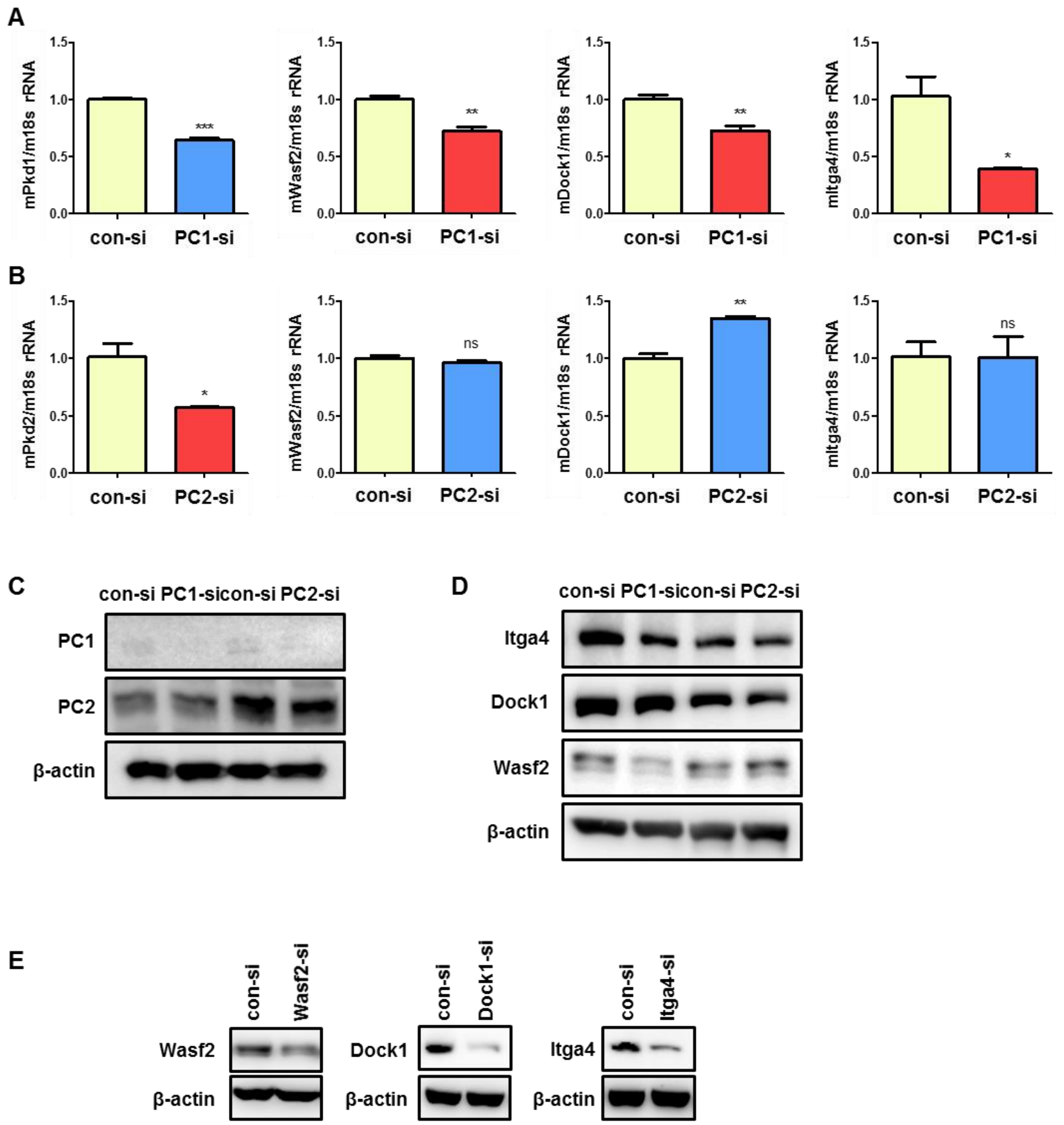

Supplementary Figure 7. Confirmation of mRNA expression levels in mIMCD spheroids. (A, B) After 6 days of mIMCD culture in matrigel, PC1/PC2 siRNA-transfected mIMCD spheroids were recovered. *Pkd1* or *Pkd2* expression level was verified and target genes (*Wasf2*, *Dock1*, and *Itga4*) of miR-182-5p were confirmed in PC1/PC2-siRNA transfected mIMCD spheroids, respectively. *, P<0.05; **, P < 0.01; ***, P < 0.001. **(C)** Protein levels of PC1 and PC2 were detected in PC1- or PC2-transfected mIMCD spheroids, respectively, by western blotting analysis. **(D)** Immunoblotting showed protein levels of Wasf2, Dock1, and Itga4 in recovered mIMCD spheroids. **(E)** Protein levels of Wasf2,

Dock1, and Itga4 observed upon transfection with Wasf2, Dock1, and Itga4-siRNA were confirmed by western blot analysis. β-Actin was used as an internal control.

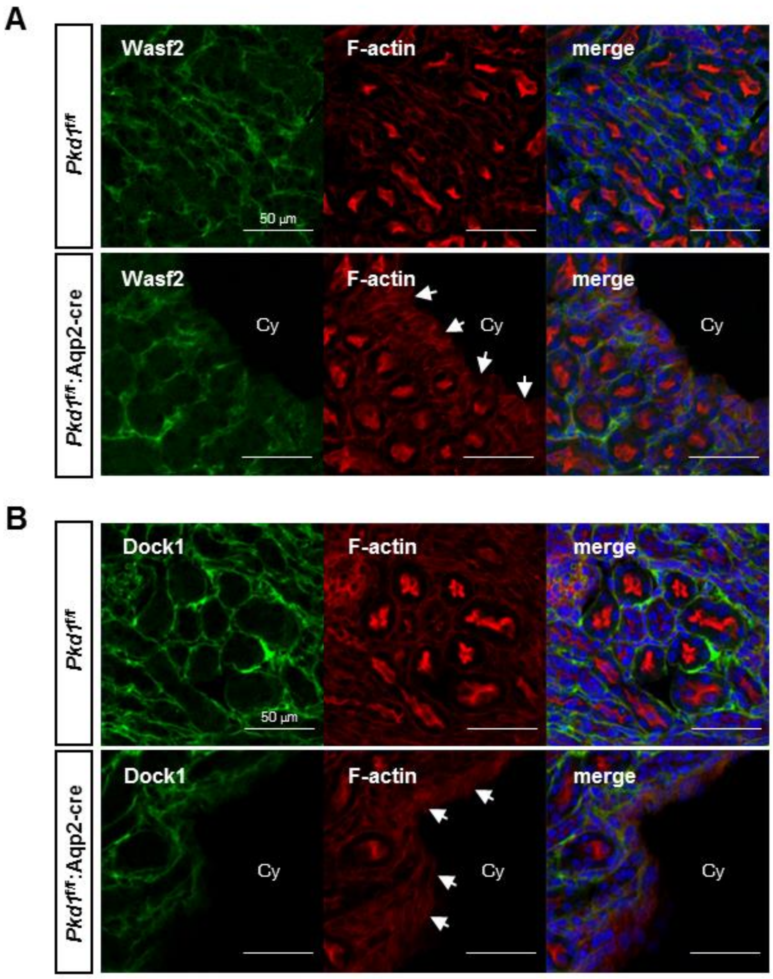

Supplementary Figure 8. *Pkd1* conditional knockout mouse models showed actin cytoskeleton defects in cyst-lining epithelial cells. (A, B) Defective actin cytoskeleton structure was observed by rhodamine-labeled phalloidin staining in *Pkd1*$^{f/f}$:Aqp2-Cre mouse kidney tissues relative to controls. Expression of Wasf2 and Dock1 in *Pkd1*$^{f/f}$:Aqp2-Cre mouse kidney tissues decreased compared to controls. Scale bars represent 50 μm. Arrows point to the cyst-lining epithelial cells. Cy, cyst.

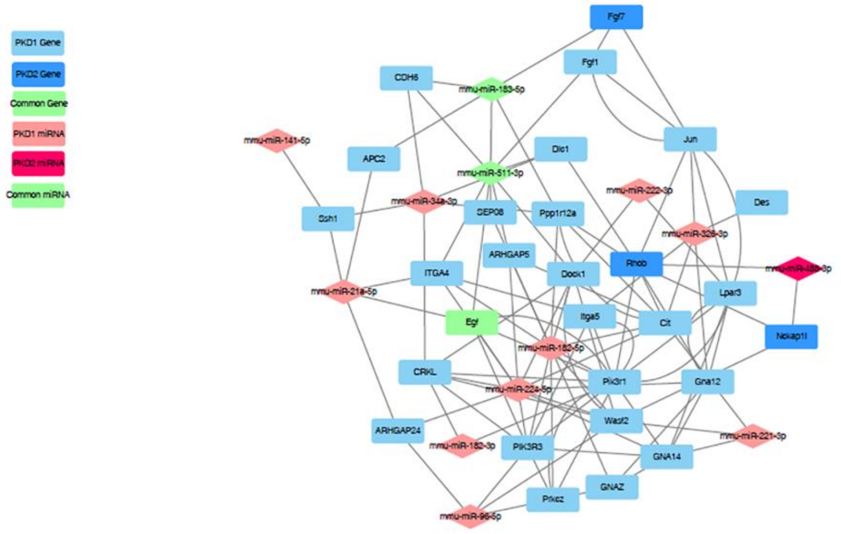

Supplementary Figure 9. Integrative network between *Pkd1* conditional knockout mice and *Pkd2* conditional knockout mice associated with the actin cytoskeleton signaling pathway.

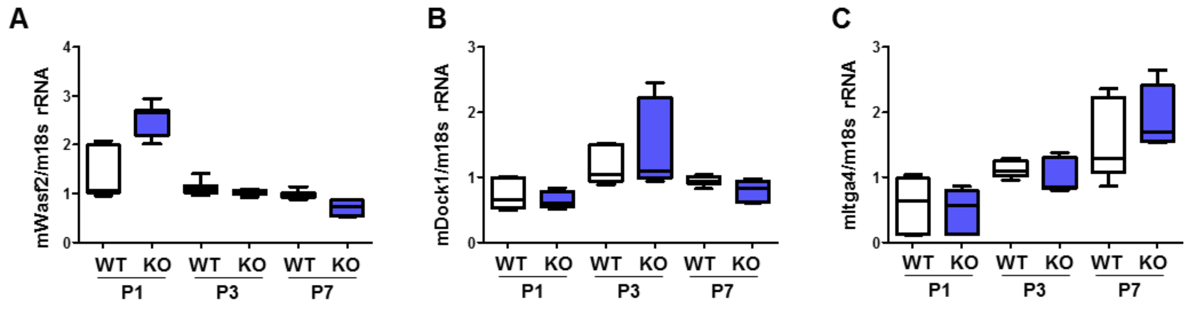

Supplementary Figure 10. Expression level of miR-182-5p target genes in *Pkd2* conditional knockout mouse kidney tissues. *Wasf2* **(A)**, *Dock1* **(B)**, and *Itga4* **(C)** mRNA expression levels were observed in *Pkd2*$^{f/f}$:HoxB7-Cre mouse kidney tissues relative to controls at postnatal days P1, P3, and P7.

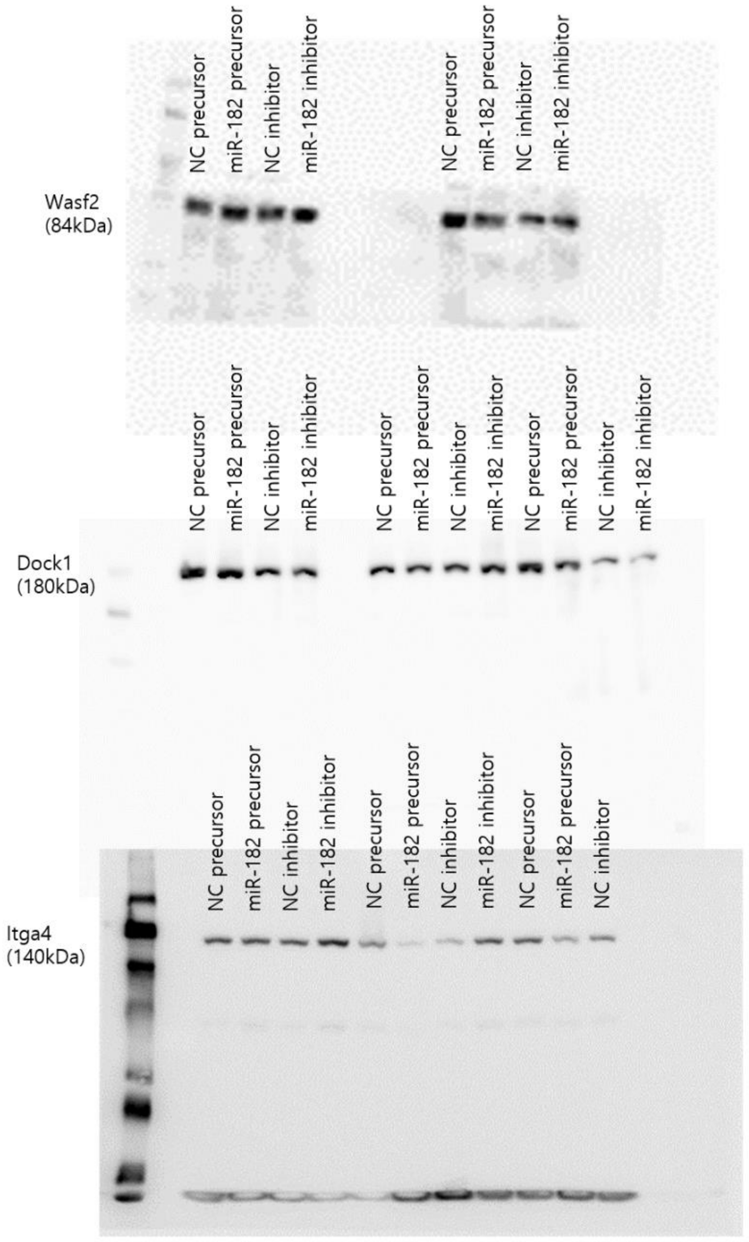

Supplementary Figure 11. The complete western blots of Figure 5A

(Supplied separately excel files)

Suppplementary Table S1. The list of differentially expressed genes (DEGs) in *Pkd1* conditional knockout mice

Suppplementary Table S2. The list of differentially expressed miRNAs (DEmiRs) in *Pkd1* conditional knockout mice

Suppplementary Table S3. The list of differentially expressed genes (DEGs) in *Pkd2* conditional knockout mice

Suppplementary Table S4. The list of differentially expressed miRNAs (DEmiRs) in *Pkd2* conditional knockout mice

Suppplementary Table S5. Ingenuity Pathway Analysis in *Pkd1* conditional knockout mice to determine biological functions and canonical pathways

Suppplementary Table S6. Ingenuity Pathway Analysis in *Pkd2* conditional knockout mice
